# Keratin 12 missense mutation induces the unfolded protein response and apoptosis in Meesmann epithelial corneal dystrophy

**DOI:** 10.1093/hmg/ddw001

**Published:** 2016-01-11

**Authors:** Edwin H.A. Allen, David G. Courtney, Sarah D. Atkinson, Johnny E. Moore, Laura Mairs, Ebbe Toftgaard Poulsen, Davide Schiroli, Eleonora Maurizi, Christian Cole, Robyn P. Hickerson, John James, Helen Murgatroyd, Frances J.D. Smith, Carrie MacEwen, Jan J. Enghild, M. Andrew Nesbit, Deena M. Leslie Pedrioli, W.H. Irwin McLean, C.B. Tara Moore

**Affiliations:** 1 School of Biomedical Sciences, University of Ulster, Coleraine BT52 1SA, Northern Ireland, UK,; 2 Centre for Dermatology and Genetic Medicine, Division of Biological Chemistry and Drug Discovery, School of Life Sciences, University of Dundee, Scotland DD1 5EH, UK,; 3 Microscopy Facility, College of Life Sciences, University of Dundee, Dundee DD1 5EH, UK,; 4 Cathedral Eye Clinic, Academy Street, Belfast BT15 1ED, UK,; 5 Department of Molecular Biology and Genetics,; 6 Interdisciplinary Nanoscience Center (iNANO) and Center for Insoluble Protein Structures (inSPIN), Science Park, Aarhus University, Aarhus, Denmark and; 7 Department of Ophthalmology, Ninewells Hospital and Medical School, Dundee DD1 9SY, UK

## Abstract

Meesmann epithelial corneal dystrophy (MECD) is a rare autosomal dominant disorder caused by dominant-negative mutations within the *KRT3* or *KRT12* genes, which encode the cytoskeletal protein keratins K3 and K12, respectively. To investigate the pathomechanism of this disease, we generated and phenotypically characterized a novel knock-in humanized mouse model carrying the severe, MECD-associated, K12-Leu132Pro mutation. Although no overt changes in corneal opacity were detected by slit-lamp examination, the corneas of homozygous mutant mice exhibited histological and ultrastructural epithelial cell fragility phenotypes. An altered keratin expression profile was observed in the cornea of mutant mice, confirmed by western blot, RNA-seq and quantitative real-time polymerase chain reaction. Mass spectrometry (MS) and immunohistochemistry demonstrated a similarly altered keratin profile in corneal tissue from a K12-Leu132Pro MECD patient. The K12-Leu132Pro mutation results in cytoplasmic keratin aggregates. RNA-seq analysis revealed increased chaperone gene expression, and apoptotic unfolded protein response (UPR) markers, CHOP and Caspase 12, were also increased in the MECD mice. Corneal epithelial cell apoptosis was increased 17-fold in the mutant cornea, compared with the wild-type (*P* < 0.001). This elevation of UPR marker expression was also observed in the human MECD cornea. This is the first reporting of a mouse model for MECD that recapitulates the human disease and is a valuable resource in understanding the pathomechanism of the disease. Although the most severe phenotype is observed in the homozygous mice, this model will still provide a test-bed for therapies not only for corneal dystrophies but also for other keratinopathies caused by similar mutations.

## Introduction

Corneal dystrophies are a group of blinding heritable conditions, with a prevalence of approximately one in 2000 (1). They are non-inflammatory conditions restricted to the cornea and usually result in a loss of corneal transparency (2). They have been classified into four separate anatomically defined groups: epithelial and subepithelial dystrophies, epithelial–stromal transforming growth factor beta-induced (TGFBI) dystrophies, stromal dystrophies and endothelial dystrophies (3). Genetic analysis of familial cases has revealed corneal dystrophic mutations arising in *TGFBI*, *KRT3*, *KRT12*, *TACSTD2*, *CHST6*, *UBIAD1*, *DCN*, *PIKFYVE*, *COL8A2*, *COL17A1*, *ZEB1* and *SLC4A11* genes (3,4).

Meesmann epithelial corneal dystrophy (MECD) is a rare autosomal dominant hereditary disorder of the anterior corneal epithelium. Typically, MECD is characterized by intra-epithelial microcysts in the central cornea, with occasional cases exhibiting grey serpiginous lines. In most cases, MECD remains asymptomatic and is only detected in routine eye tests. However, it can become more debilitating with some individuals experiencing photophobia, blurred vision or foreign body sensation with rupture of epithelial cysts at the corneal surface. MECD is caused by heterozygous mutations in either the *KRT3* or *KRT12* genes, which encode corneal-specific keratins K3 and K12, respectively (5,6). These form K3–K12 heterodimers that polymerize to form the intermediate cytoskeletal filaments that provide structure and stability to corneal epithelial cells (7,8). Twenty-three mutations in *KRT12* and three in *KRT3* have been reported to cause MECD of variable severity (www.interfil.org, October 2015, date last accessed). Apart from one small in-frame insertion, all of the causative variants are missense and are known to exert a dominant-negative effect (6). All mutations are located in the functionally critical helix initiation or helix termination motifs, indicating that protein misfolding plays a role in MECD (9). However, the mechanism by which K12 mutations cause corneal microcyst formation and cytolysis remains poorly understood.

The unfolded protein response (UPR) is a pathway stimulated to respond to an accumulation of misfolded or mutant proteins within the endoplasmic reticulum (ER) (10). Proteins such as chaperones and ER-associated degradation components are recruited to placate cellular protein translation (11). However, prolonged induction of the UPR because of continuous expression and misfolding of mutant protein can lead to apoptosis (10). This occurs in a variety of conditions with a genetic basis, including Alzheimer disease (12) and cystic fibrosis (13). The induction of cellular stresses in corneal disorders is not uncommon. UPR pathways have previously been shown to be induced in Fuchs endothelial corneal dystrophy (14–16) and in the formation of cataracts (17). Members of the keratin family of proteins have also been implicated in cellular stress responses, ranging from apoptotic stress and oxidative stress to wound healing and tissue repair (18,19).

To study the mechanisms of corneal fragility and cytolysis associated with MECD-causing keratin K12 mutations, we generated a targeted humanized knock-in mouse model of MECD and investigated the corneal phenotypes that developed. Although K12-Arg135Thr is the most common MECD-causing mutation found in the European population (5), the most severe phenotype has been observed in patients with the K12-Leu132Pro mutation. In these patients, subepithelial basement membrane scarring and central corneal opacification can result in blindness. Furthermore, the K12-Leu132Pro protein more readily forms aggregates than K12-Arg135Thr protein (20). Therefore, mice were generated with the human mutant *KRT12* allele carrying the Leu132Pro mutation, and a C-terminal Flag-HA epitope tag, into the mouse *Krt12* locus by homologous recombination. Histological analysis of the corneas was performed to ascertain any irregularities in the structure of the cornea, whereas immunohistochemistry (IHC) was used to visualize keratin expression profiles and assess the expression of UPR markers. These expression profiles were also assessed in corneal tissue from a *K12*-Leu132Pro MECD patient. A proteomic analysis was also performed on the human MECD cornea, and compared with control corneal tissue, to ascertain the presence of keratin dysregulation in the human phenotype.

## Results

### Generation of humanized *KRT12* mutant knock-in mice

**Figure 1.**
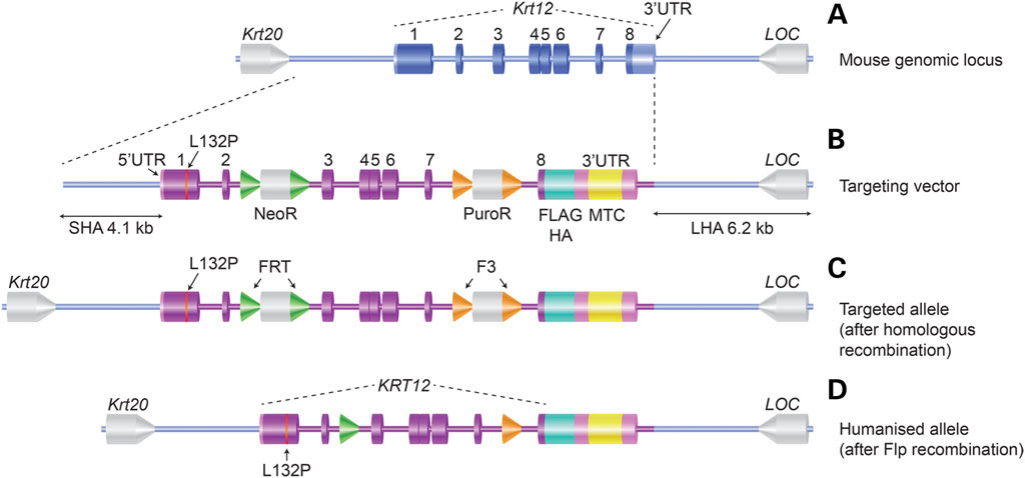
Generation of a humanized mutant K12 mouse model. The endogenous mouse *Krt12* gene in its genomic context (**A**) was targeted for homologous recombination by a vector incorporating the human *KRT12* gene (**B**). The targeting vector contained two homology arms (LHA and SHA) for targeted replacement of the endogenous mouse *Krt12* with the humanized genomic region. The human allele includes all UTRs, exons and introns with additional coding for a FLAG-HA tag immediately prior to the stop codon and a single nucleotide change in exon 1 creating the L132P mutant (B). Additionally, an MTC is included within the 3′-UTR. FRT- and F3-flanked selection markers were included within introns 2 and 7 to assist in the selection of clones where the transgene had been correctly targeted (**C**). Transgene-positive mice were finally interbred with transgenic mice universally expressing Flp recombinase to excise the selection markers (**D**). *Krt20* and *LOC* are neighbouring genes.

A targeting vector was generated that contained the full human *KRT12* gene including introns, untranslated regions (UTRs) and a 247 bp sequence downstream of the *KRT12* 3′-UTR. Exon 1 carried the L132P mutation, and a FLAG-HA coding sequence (21) was placed immediately before the termination codon. A multiple targeting cassette (MTC) was inserted into the *KRT12* 3′-UTR, consisting of target sequences for four MECD mutations (Supplementary Material, Fig. S1). The targeting strategy is shown in Figure 1. This targeting vector was used to introduce the human Leu132Pro *KRT12* mutant allele (*KRT12-L132P*) into the murine *Krt12* locus by homologous recombination in mouse embryonic stem cells, which were used to generate transgenic mice on the C57BL/6 background (Fig. 1). Germline transmission of the targeted allele was confirmed by polymerase chain reaction (PCR) (Supplementary Material, Fig. S4), and recombinant mutant mice were bred with transgenic C57BL/6 mice universally expressing Flp-deleter recombinase to excise the Neo^R^ and Puro^R^ cassettes (Fig. 1) (22). The resultant *KRT12-L132P-*positive, Neo^R^/Puro^R^-negative mouse line was designated *Krt12*^hL132P^. Heterozygous (*Krt12*^+/hL132P^) and homozygous (*Krt12*^hL132P/hL132P^) mutant mice were viable and fertile with no superficial corneal abnormalities (data not shown).

### Characterization of heterozygous (*Krt12*^+/hL132P^) and homozygous (*Krt12*^hL132P/hL132P^) corneal epithelium

Mice of each genotype [wild-type (WT), heterozygous, homozygous; *n* = 6] were assessed for gross changes in corneal morphology by slit-lamp evaluation at 8, 12, 16 and 30 weeks. No differences were observed with respect to the clarity of the cornea between the genotypes (Supplementary Material, Fig. S2A). Additionally, at 16 weeks, mouse eyes were observed following fluorescein staining to highlight areas of damage or abnormality on the corneal surface. Again, no overt differences were observed between the genotypes (Supplementary Material, Fig. S2B).

### Histology and electron microscopy of *Krt12*^+/hL132P^ and *Krt12*^hL132P/hL132P^ corneal epithelium

**Figure 2.**
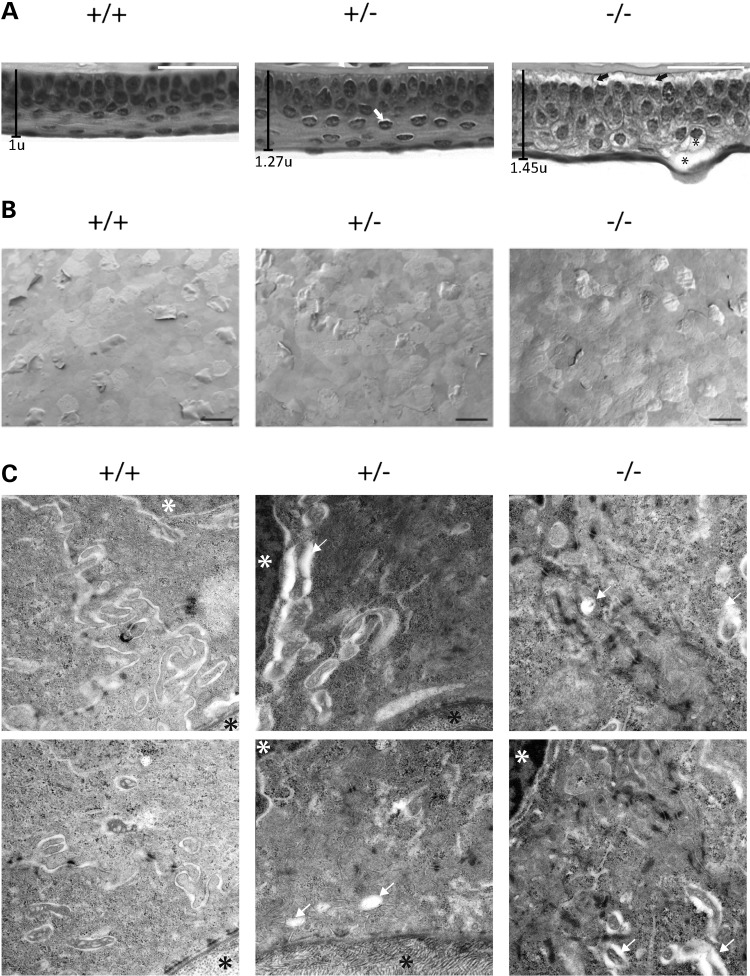
Microscopic assessment of MECD mouse corneas. (**A**) Representative images of H&E-stained 8-week-old mice corneas. WT mice typically have a tightly packed corneal epithelium, with subtle small intracellular spaces occasionally visible in the heterozygous mice (white arrows). Homozygous mutant mice exhibited greater phenotypic changes, with cells less tightly packed and with large intracellular spaces leading to cytolysis (asterisks); delamination of the corneal epithelium at the stromal interface (black arrows) was observed in the homozygous mutant mice. Scale bar = 100 µm. (**B**) SEM micrographs of the corneal surface (*n* = 1 per genotype, both eyes) show squames flaking or beginning to flake off; however, no significant differences were observed on the corneal surface between genotypes (scale bar, 50 µm). (**C**) TEM micrographs of the basal layer cells of each genotype (25 000× magnification) show the stroma (black asterisks) and nuclei (white asterisks). WT cells appear to have a more distinct structure with tight cell–cell interfaces in comparison to homozygous mutant cells. Vacuoles and structures associated with mechanical weakness (white arrows) are observed in these cells. The corneas of heterozygous mutant more closely resembled those of the WT mice; however, some evidence of an increase in vacuolization was noted (white arrows).

Whole eyes were harvested from mice (*n* = 3 per genotype) at 8, 16 and 24 weeks. The observed histology did not change significantly over time and representative images of 8-week-old mice are shown (Fig. 2). The corneal epithelium of WT animals showed a close packed, regular cell structure; however, the corneal epithelial cells of homozygous mutant mice were highly disorganized (Fig. 2A). Additionally, although the number of cell layers was the same for each genotype, the corneal epithelium was ∼50% thicker in homozygous mutant animals (Fig. 2A). Mutant cells were larger, contained prominent intracellular spaces and showed overt cytolysis with occasional cell rupture at the corneal surface. Delaminations at the basal layer in the homozygous animals were also observed, reminiscent of structural defects seen in the K12 null mouse (23). Defects in heterozygous mice were very subtle; however, intracellular spaces were clearly increased. Additionally, no cytolysis or delamination of the corneal epithelial interface was observed in heterozygous mice (Fig. 2A).

The corneal surfaces of 7-week-old mice (*n* = 1 per genotype) were examined by scanning electron microscopy (SEM). Sloughing of terminally differentiated epithelial cells was observed in all genotypes. However, no corneal surface defects were observed in mutant mice (Fig. 2B). These corneas were also evaluated by transmission electron microscopy (TEM). WT corneal keratinocytes showed normal cellular structure. In contrast, keratinocytes from homozygous mutant mice showed cytoplasmic vacuoles and other defects suggestive of cell fragility (Fig. 2C), with similar but subtle changes present in heterozygous mice.

### IHC analysis of transgene expression

**Figure 3.**
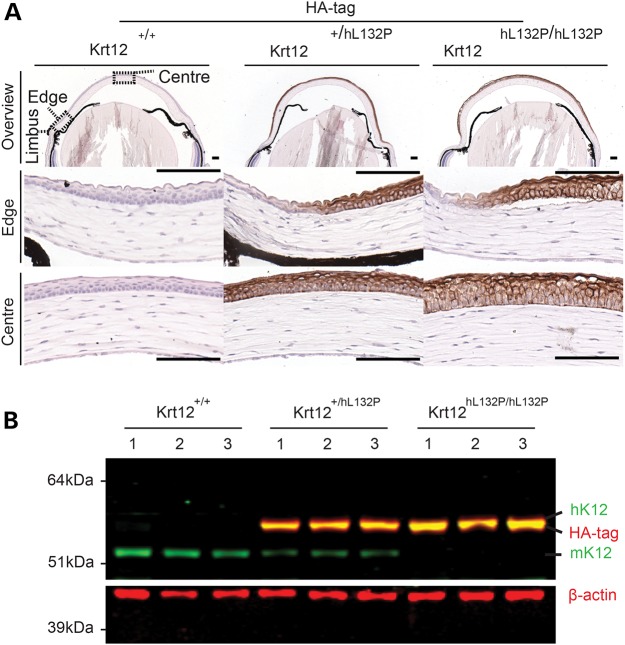
Verification of *KRT12* transgene expression. (**A**) The *KRT12* transgene has a C-terminal FLAG-HA epitope tag. The HA epitope is present only in the heterozygous and homozygous mutant mice (brown staining). Staining was observed in all epithelial layers moving centrally and inward from the limbal region (scale bar, 100 µm). (**B**) Corneal protein lysates from 7 to 9-week-old mice (*n* = 3 per genotype) were simultaneously probed for K12 and HA. Only mK12 was observed in corneas from WT mice (green lower band), whereas only the HA-tagged protein (green hK12 and red HA co-localize for a yellow merged image) was present in homozygous mutant mice. Both proteins were present in heterozygous mice. β-actin was used as a loading control.

The humanized *KRT12-L132P* transgene has a C-terminal FLAG-HA epitope tag, which enabled IHC confirmation of transgene expression and translation in heterozygous and homozygous mice (Fig. 3). Expression was specific to the corneal epithelium and observed in all epithelial layers moving centrally from the limbal region. Western blotting confirmed transgene expression in heterozygous and homozygous animals (Fig. 3).

### Gene expression analysis in Krt12^hL132P^ mouse cornea

**Figure 4.**
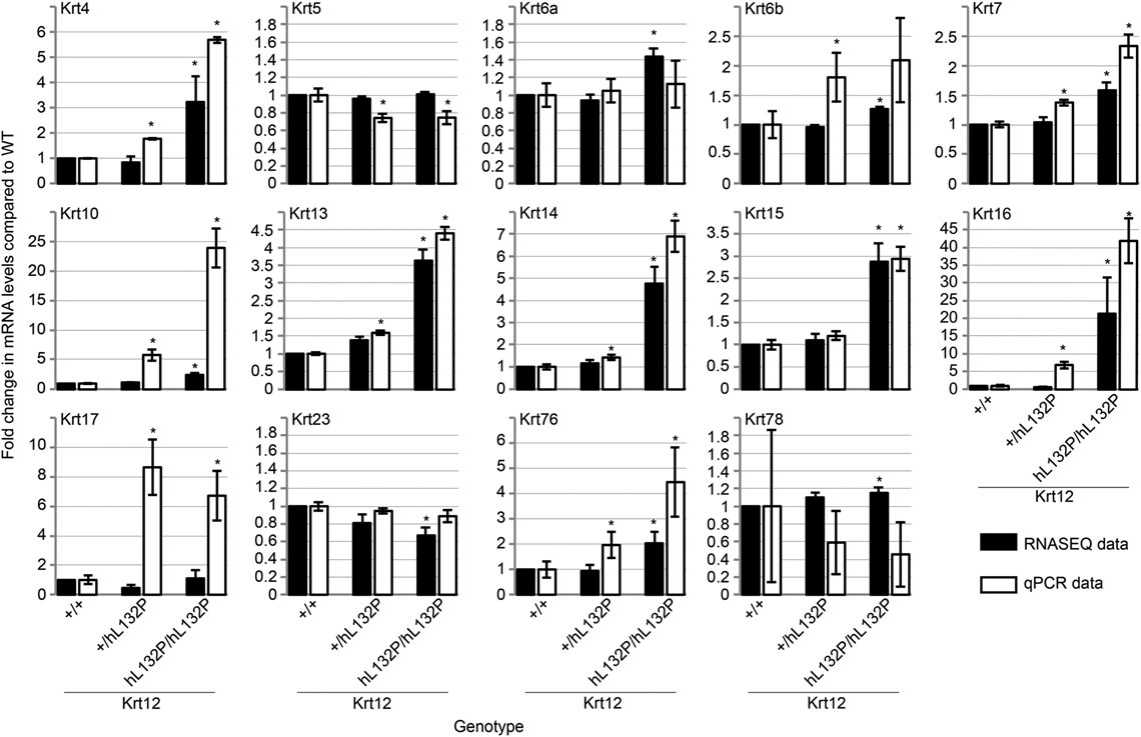
Differential expression of keratin mRNA in MECD mouse corneas. qRT-PCR validation of keratin mRNA levels obtained by RNA-seq. Fold change in expression for each keratin was calculated by normalization to that observed in the WT mouse cornea. Similar expression levels were observed for RNA-seq and qRT-PCR in all keratins analysed except KRT10 and KRT17, in which greater expression was found in heterozygous and homozygous mice by qRT-PCR, whereas RNA-seq deemed these keratins to be comparably expressed in all three genotypes.

mRNA from WT, heterozygous and homozygous Krt12^hL132P^ mutant mice was isolated from mouse corneas (*n* = 6 per group) and gene expression quantified by RNA-seq analysis. Sequencing data were analysed by comparing mRNA expression in the cornea of heterozygote or homozygote mutant mice to the WT reference (*n* = 6 corneas per group). The genes with statistically significant changes in expression, adjusted for false discovery rate (*P* < 0.05), were determined. The most dysregulated genes, with respect to the WT, in the heterozygous (two genes) and homozygous mutant (25 genes) mice were tabulated and sorted by fold change (Supplementary Material, Table S1). Ten of the most highly upregulated genes were lens crystallins, which showed increases in expression between 8.4- and 520-fold. In the homozygous *Krt12*^hL132P/hL132P^ cornea, the expression of 11 keratin genes was upregulated (Fig. 4). These data were verified by quantitative real-time (qRT)-PCR (Fig. 4). *Krt16* was the most highly upregulated keratin mRNA (approximately 20-fold). *Krt14* was upregulated approximately 5-fold with *Krt4*, *Krt10*, *Krt13* and *Krt15* upregulated approximately 3-fold. *Krt6a*, *Krt6b*, *Krt7*, *Krt76* and *Krt78* were also upregulated but to a lesser extent (less than 2-fold). qRT-PCR analysis showed that *Krt5* was downregulated (approximately 0.5-fold). qRT-PCR analysis of keratin gene expression in heterozygous *Krt12*^+/hL132P^ cornea showed an alteration similar to that seen in *Krt12*^hL132P/^^hL132P^, with smaller, but significant, alterations in expressions of *Krt4*, *Krt5*, *Krt6b*, *Krt7*, *Krt14*, *Krt16*, *Krt17* and *Krt76* (0.7–9-fold) (Fig. 4).

### IHC analysis of corneal keratin protein expression patterns

**Figure 5.**
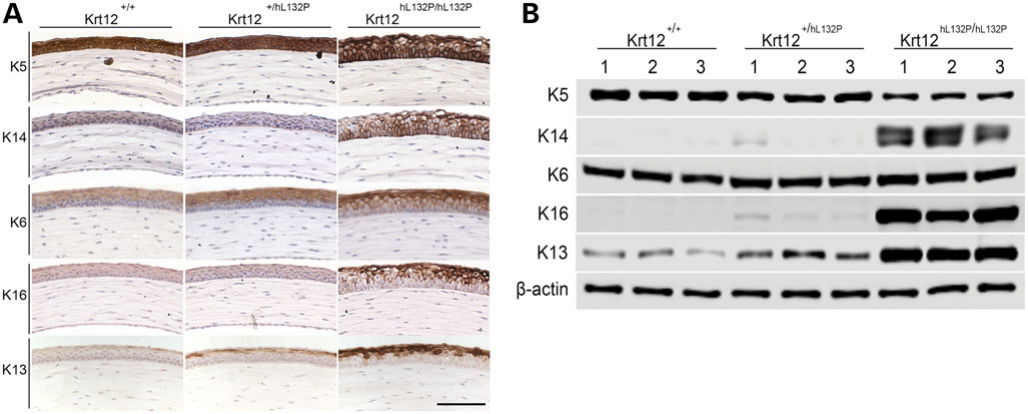
Differential expression of keratins in MECD mouse cornea. (**A**) Representative pictures of IHC staining (brown, nuclei blue) of the central corneas of 8-week-old mice are shown. K5 was present throughout all layers of the corneal epithelium in all genotypes. Expression of K14 was markedly increased in the homozygous animals. K6 was found in the outer layer of all corneas with staining significantly increased in the homozygous mutant; K16 was also strongly upregulated in homozygous mutant mice. Scale bar, 100 µm. (**B**) Immunoblotting of corneal lysates (*n* = 3 per genotype) for keratins K5, K14, K6, K16 and K13, normalized to β-actin. K5 levels were greatly reduced when WT and heterozygous mice and heterozygous and homozygous mutant animals are compared. K14 was strongly upregulated in homozygous mouse cornea, with a small change in heterozygous mice. K6 increased with increasing transgene expression when WT and homozygous mice are compared. Similar changes in expression pattern were observed with K16 and K13.

To verify the altered keratin expression observed by RNA-seq and qRT-PCR analyses, corneal keratin expression was assessed by IHC (*n* = 2 per genotype at 8, 12 and 16 weeks; Fig. 5A) and validated by western blotting (Fig. 5B; *n* = 3 per genotype, mice aged 7–9 weeks). Figure 5A shows representative IHC data from 8-week-old mice, in which keratin expression changes were clearly observed. Identical results were obtained at the other time points (data not shown). With no murine equivalent to K3, K5 has been shown to be the endogenous polymerization partner of K12 in murine cornea (7). Immunohistological analysis revealed K5 to be present within all layers of the cornea in all genotypes. However, western blot analysis, in agreement with the qRT-PCR data, showed that K5 abundance decreased proportionally with reduced WT mK12 expression, with K5 expression in homozygous mutant mice less than half that of the WT mouse (*P* < 0.05; Fig. 5B and Supplementary Material, Fig. S3).

The heterodimeric partner of K5 in skin is K14, in both humans and mice (24). In response to reduced mK12 expression, K14 mRNA expression was strongly upregulated in homozygous mutant animals, with an approximate 19-fold increase in protein expression observed by western blot analysis of homozygous mutant mouse corneas (*P* < 0.05; Fig. 5B and Supplementary Material, Fig. S3).

K6 was found in the upper suprabasal layers of WT mice (Fig. 5A), and its expression was greatly increased in homozygous mutant mice and was confirmed by western blot analysis with an approximate 1.5-fold increase (*P* < 0.05; Fig. 5B and Supplementary Material, Fig. S3). IHC and western blot analyses also showed that expression of K16, a heterodimeric partner of K6, was upregulated by almost 1000-fold in homozygous mutant mice (*P* < 0.05). Additionally, no expression of the conjunctival keratins K8 and K19 was observed in the corneal epithelium of any of the genotypes (data not shown).

### Histological and immunohistological examination of human Leu132Pro *KRT12* MECD corneal tissue

To examine whether the changes in the keratin expression profile seen in the mouse model reflect changes seen in human MECD cornea, a histological and IHC analysis was performed on a cornea obtained, following corneal graft surgery, from an MECD patient with the same heterozygous Leu132Pro *KRT12* mutation.

**Figure 6.**
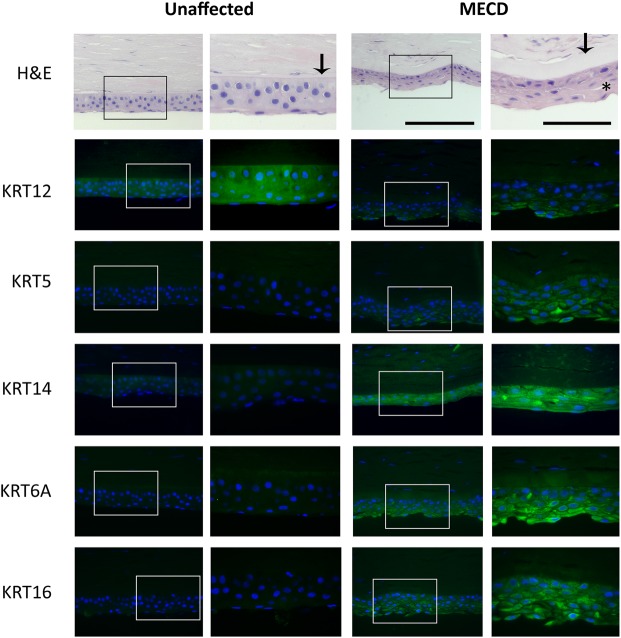
Differential expression of keratins in the human MECD corneal epithelium. Histological assessment of the human MECD cornea demonstrated a loss in epithelial organization in comparison to the healthy unaffected cornea. Sections from both corneas were also assessed by IHC for the expressions of K12, K5, K14, K6A and K16 (green). Nuclei were counterstained with DAPI (blue). The MECD tissue appeared to have a decreased abundance of K12 protein in the epithelium, compared with the control. Expression of K5, K14, K6A and K16 was increased in the MECD cornea, compared with that found in the control cornea. Scale bar = 100 and 200 µm, left and right images, respectively.

Histology of the MECD cornea revealed characteristic disorganization of the corneal epithelium and a pronounced thickening of Bowman's layer in comparison to the healthy control tissue (Fig. 6, black arrow). The appearance of microcysts and intra-cytoplasmic vacuoles, similar to those previously reported in MECD corneal tissue (5) (Fig. 6, represented by an asterisk), was also noted. IHC analysis of keratin expression revealed patterns of altered expression in the human MECD corneal epithelium similar to those observed in the MECD mouse model, with increased expressions of K6, K14 and K16. In contrast to the mouse, and perhaps reflecting that in humans the heterodimeric partner of K12 is K3, K5 expression is increased in parallel to its human heterodimeric partner, K14 (Fig. 6). IHC also revealed a reduced expression in K12 in the MECD cornea, in comparison to the control healthy corneal tissue.

### Determination of the MECD corneal proteome

To further characterize the changes in protein expression occurring in the cornea of MECD patients, we undertook an analysis of the corneal proteome of one MECD patient using liquid chromatography–tandem MS.

Upon assessment of the healthy corneal tissue and MECD corneal tissue, a list of the most abundant proteins present within each tissue was constructed and their relative abundances calculated. Proteins were displayed as their molar percentage within the sample (Supplementary Material, Table S2), and the 25 most abundant proteins in each tissue determined (Table 1). Reflecting that this analysis represented the proteome of the whole cornea, the expression profiles of the normal and diseased corneas were broadly similar, with the expression of stromal extracellular matrix proteins such as collagens [alpha-1(I), alpha-2(I), alpha-1(IV) and alpha-3(VI)], proteoglycans (keratocan, decorin and lumican) and corneal epithelial chaperone proteins (aldehyde dehydrogenase) unaltered. Notably, and in agreement with the IHC analysis, there were increases in the relative abundance of keratins K5, K14, K6A and K13 (Table 2). However, an increase in K12 was also found, in contrast to that observed by IHC of the human MECD cornea.

**Table 1.** Relative amounts of the 25 most abundant proteins in control and MECD human corneal tissues

Control tissue			MECD tissue		
	Name	Amount (%)		Name	Amount (%)
1	Serum albumin	12.28	1	Collagen alpha-1(I) chain	9.50
2	Collagen alpha-1(I) chain	10.72	2	Serum albumin	9.07
3	Collagen alpha-2(I) chain	5.32	3	Collagen alpha-2(I) chain	4.06
4	Ig kappa chain C-region	5.06	4	Ig kappa chain C-region	3.48
5	TGFBI	4.28	5	TGFBI	2.86
6	Keratocan	3.23	6	Histone H4	2.64
7	Vimentin	2.53	7	Keratin, type II cytoskeletal 5	2.61
8	Decorin	2.35	8	Keratocan	2.41
9	Ig lambda-2 chain C regions	2.33	9	Ig lambda-2 chain C regions	2.41
10	Collagen alpha-3(VI) chain	2.06	10	Histone H2B type 1-B	2.15
11	Lumican	2.02	11	Keratin, type I cytoskeletal 14	2.10
12	Aldehyde dehydrogenase, dimeric NADP-preferring	1.86	12	Lumican	2.06
13	Mimecan	1.85	13	Histone H2B type 1-C/E/F/G/I	1.93
14	Ig gamma-1 chain C-region	1.83	14	Keratin, type II cytoskeletal 6A	1.92
15	Histone H2B type 1-H	1.68	15	Collagen alpha-3(VI) chain	1.80
16	Histone H2B type 1-C/E/F/G/I	1.66	16	Decorin	1.76
17	Serotransferrin	1.38	17	Apolipoprotein A-I	1.73
18	Apolipoprotein A-I	1.25	18	Haemoglobin subunit beta	1.55
19	Histone H4	1.23	19	Ig gamma-1 chain C-region	1.52
20	Apolipoprotein A-II	1.22	20	Aldehyde dehydrogenase, dimeric NADP-preferring	1.40
21	Collagen alpha-1(VI) chain	1.14	21	Keratin, type I cytoskeletal 13	1.37
22	Prolargin	1.13	22	Apolipoprotein A-II	1.34
23	Ig kappa chain V-III region SIE	1.04	23	Collagen alpha-1(VI) chain	1.28
24	Annexin A2	0.96	24	Keratin, type I cytoskeletal 12	1.27
25	Ig gamma-3 chain C-region	0.95	25	Vimentin	1.17

A list of the top 25 proteins, by percentage abundance, found in healthy control or MECD corneal tissue was produced. Keratin proteins were only found in the 25 most abundant proteins in the MECD corneal tissue. Results are representative of one MECD patient cornea and one healthy control patient cornea.

**Table 2.** Comparison of keratins with >1% abundance in control or MECD tissue

	Relative abundance (%)		Fold change
Name	Control	MECD	
Keratin, type II cytoskeletal 5	0.53	2.61	+4.9
Keratin, type I cytoskeletal 14	0.13	2.10	+15.6
Keratin, type II cytoskeletal 6A	0.00	1.92	
Keratin, type I cytoskeletal 13	0.00	1.37	
Keratin, type I cytoskeletal 12	0.15	1.27	+8.4

In total, 10 keratin proteins were found across both corneal tissue samples. For keratins with a relative abundance >1% in either tissue and found in both tissues, the fold change in abundance between MECD and control tissues was calculated.

### UPR activation and apoptosis in the *Krt12*^+/hL132P^ and *Krt12*^hL132P/hL132P^ corneal epithelium

Missense mutations in *Krt12* have been demonstrated to result in the accumulation of protein aggregates in corneal epithelial cells, presumably due to protein misfolding (20,25). Accumulation of misfolded or mutant proteins within both the cytoplasm and the ER stimulates the UPR, which, if it fails to resolve the ER stress, may in turn lead to increased apoptosis (15).

**Figure 7.**
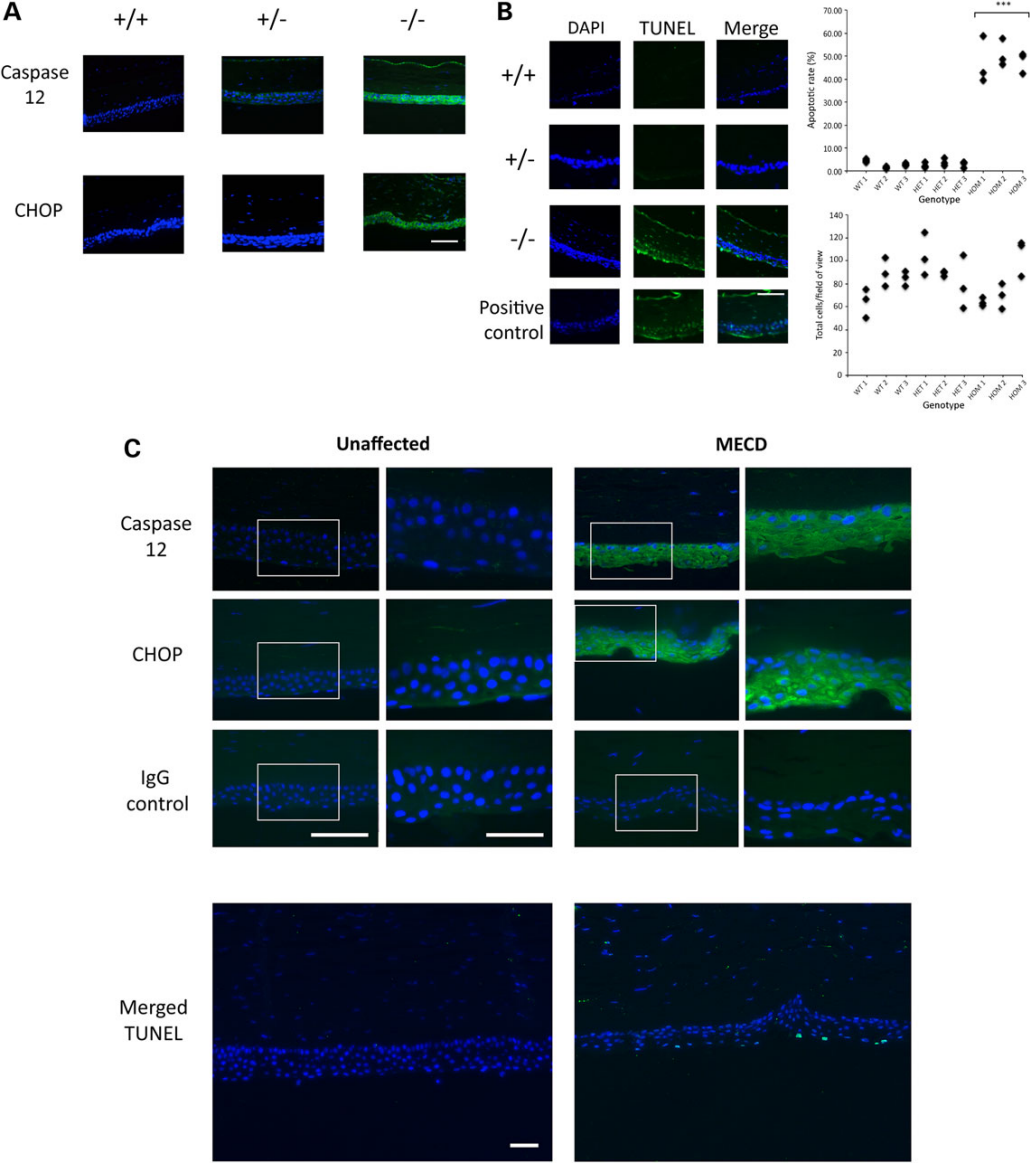
Assessment of induction of the UPR and apoptosis in MECD mouse corneas. (**A**) IHC analysis of UPR markers, Caspase 12 and CHOP, was performed on corneal sections from WT (+/+), heterozygous (+/−) and homozygous MECD mice (green). Nuclei were counterstained with DAPI (blue). Scale bar = 100 µm. Positive staining for both UPR markers was observed in the homozygous mouse corneal epithelium, whereas subtle staining of Caspase 12 was found in the corneal epithelium of heterozygous mice. (**B**) A TUNEL assay was performed to identify the presence of apoptotic cells within the corneas of these mice (green). The number of TUNEL-positive cells was counted to ascertain the apoptotic cells, whereas DAPI-stained cells were counted to gauge the total number of cells. A significant increase in the proportion of apoptotic cells was observed in homozygous mice when compared with WT, with the rate in heterozygous mice being comparable to WT. Scale bar = 100 µm.

We sought to determine whether expression of mutant K12 in corneal epithelial cells activated the UPR pathway and apoptosis. ER stress-driven stimulation of the UPR is associated with the induction of CCAAT/enhancer-binding protein homologous protein (CHOP) expression (26). IHC analysis of corneal sections from 24-week-old WT, heterozygous and homozygous mutant mice showed that expression of CHOP was upregulated in the corneal epithelium of homozygous *Krt12*^hL132P/hL132P^ mice but not in either heterozygous *Krt12*^+/hL132P^ or WT *Krt12*^+/+^ mice (Fig. 7A).

Failure of the UPR to resolve ER stress results in the stimulation of caspase-mediated apoptotic pathways (27,28). We investigated the induction of Caspase 12, as this protein has previously been shown to be activated during UPR-induced apoptosis in a corneal dystrophy mouse model (15). IHC analysis showed upregulation of Caspase 12 in the cornea of homozygous *Krt12*^hL132P/hL132P^ mice and, to a lesser extent, in heterozygous *Krt12*^+/hL132P^ mice at 24 weeks of age, but not in WT *Krt12*^+/+^ mice (Fig. 7A).

Analysis of corneal sections using a terminal deoxynucleotidyl transferase dUTP nick end labelling (TUNEL) assay (29) showed increased apoptosis in the corneas of homozygous *Krt12*^hL132P/hL132P^ mice at 24 weeks of age when compared with either heterozygous *Krt12*^+/hL132P^ or WT *Krt12*^+/+^ mice (Fig. 7B). The cellularity and apoptotic rate observed in corneal epithelial cells were quantitated (Fig. 7B). The average number of live cells/field-of-vision for WT, heterozygous and homozygous mice was 80, 91 and 80, respectively, with no significant difference between any of the genotypes. However, the average rate of apoptosis was significantly increased (*P* < 0.001) in homozygous *Krt12*^hL132P/hL132P^ mice (48.1 ± 6.64%) compared with either heterozygous *Krt12*^+/hL132P^ (2.8 ± 1.32%) or WT *Krt12*^+/+^ mice (2.8 ± 1.47%) (Fig. 7B). These results suggest that the UPR is able to resolve the ER stress induced by mutant L132P K12 in the heterozygous *Krt12*^+/hL132P^ mice but not in the homozygous *Krt12*^hL132P/hL132P^ mice.

### UPR in the MECD human cornea

To examine whether the stimulation of the UPR and apoptosis seen in the mouse model reflect changes seen in human MECD cornea, IHC and TUNEL analyses were performed on a cornea obtained from an MECD patient heterozygous for the dominant-negative L132P *KRT12* mutation and compared to a cornea from an unaffected donor eye. Although no expression of either CHOP or Caspase 12 was observed in the unaffected cornea, expression of both was found in the corneal epithelium of the MECD cornea (Fig. 7C), suggesting an upregulation of the UPR. Although a small number of apoptotic cells was observed by TUNEL staining of the human MECD corneal section, and none in the unaffected cornea (Fig. 7C), corneal material from additional MECD patients was unavailable to determine whether this difference was statistically significant.

## Discussion

We have generated a mouse model for MECD and demonstrated that replacement of the WT mouse *Krt12* allele with of the human *KRT12*-Leu132Pro mutant allele results in a corneal phenotype that closely resembles that of MECD patients heterozygous for mutation in *KRT12* (2,9,20). These patients were described to have opacities in the corneal epithelium caused by intra-epithelial cysts. This model demonstrates that the *KRT12* Leu132Pro mutation results in a disorganized corneal epithelium with cell fragility, delamination of the basal layer and overt cytolysis generating cysts that occasionally rupture at the corneal surface. This is the first MECD model to carry a mutation within the *KRT12* gene that is known to cause MECD in man and as such will provide an *in vivo* model in which to test potential therapies for epithelial corneal dystrophies.

Two mouse models of altered *Krt12* have been reported, and neither presents with a phenotype that closely matches that of MECD. A *Krt12* knock-out mouse has been developed, in which heterozygous mutant mice have a normal phenotype and homozygous null mice exhibit corneal epithelial fragility and mild corneal epithelial erosion (23). Unlike the Leu132Pro model, the number of cell layers in the corneal epithelium of null mice is reduced. The superficial epithelial cells of *Krt12* null mice lack keratin intermediate filaments and are easily detached by gentle rubbing. The lack of severe epithelial defects such as corneal ulceration and perforation in the *Krt12* null mice was attributed to the rapid replacement of lost superficial epithelial cells from deeper layers. This may also explain the lack of a phenotype as severe as that seen in patients with Leu132Pro *KRT12* mutation in the mice with the same mutation.

Bi-allelic expression of *Krt12* and Cre recombinase in a second mouse *Krt12* model was used to examine the possibility of clonal allelic activation in corneal epithelial cells (30). Random activation of one or the other *Krt12* alleles was observed during the course of terminal corneal epithelial cell differentiation. The authors suggested that this selection could be advantageous, as it would allow the generation of cells expressing haplosufficient quantities of WT K12 in the absence of dominant-negative mutant K12. However, uniform expression of HA-tagged Leu132Pro mutant K12 protein in all cells of the corneal epithelium of heterozygous *Krt12*^+/hL132P^ mice suggests that this is not the case.

These differences suggest that unlike the *Krt12* null mouse, our *KRT12* Leu132Pro mouse appears to represent a highly relevant model in which to study the pathogenesis of epithelial corneal dystrophy caused by mutations in *KRT12*. However, despite the similarities in phenotype observed in heterozygous MECD patients and homozygous *Krt12*^hL132P/hL132P^ mice, some differences should be noted. In contrast to the *Krt12*^hL132P/hL132P^ mice, slit-lamp photography of *KRT12* Leu132Pro MECD patients reveals, in addition to characteristic microcysts in the anterior epithelium, an uneven corneal topography secondary to damage and scarring of the underlying basement membrane and anterior stroma (20). The phenotype is even milder in the directly genotype comparable heterozygous *Krt12*^+/hL132P^ mice.

K12 is a type I (acidic) keratin produced by differentiated corneal epithelial cells that heterodimerises in a parallel, in-register fashion with K3, a type II (basic keratin). These heterodimers associate to form a number of species of stable tetramer, which ultimately polymerise form the ∼10nm diameter keratin intermediate filaments (31–33). The structures of both type I and type II keratins consist of an approximately 300 amino acid central rod domain consisting of four alpha-helical segments separated by three flexible linker domains. At the beginning of the first and at the end of the last helical domain are the highly conserved approximately 20 amino acid helix initiation and termination motifs, respectively. These, in turn, are flanked by the N-terminal head and C-terminal tail domains, which are the most divergent domains between different keratins and are thought to confer tissue-specific functions such as interactions with other cytoplasmic or membrane proteins. All known MECD mutations in *KRT3* and *KRT12* consist of dominant-negative missense mutations (or one small in-frame insertion) that occur in either the helix initiation or the termination motifs (www.interfil.org, 15 October 2015, date last accessed) that fulfil a critical role in the early stages of filament assembly (34). Mutations in these regions in type I and type II keratins are associated with the most severe disease phenotypes (6,35). Failure to adopt the correct secondary or tertiary structure or to assemble into keratin filaments is likely to result in protein misfolding and/or aggregate formation and induction of the UPR. Formation of keratinous cytoplasmic aggregates has been observed in cells transfected with Leu132Pro KRT12 (20) and in transmission electron micrographs of corneal epithelial cells from an MECD patient (25).

Although translated in the cytoplasm, accumulation of misfolded proteins such as keratins can induce ER stress and the UPR (36). The first response of cells to the accumulation of unfolded proteins is to reduce global protein translation to reduce the ER protein load and the upregulation of cytoplasmic molecular chaperones, such as heat shock proteins (HSPs), which act to restore protein homeostasis (proteostasis) (10). Originally described to maintain transparency of the lens, crystallins have a protective influence on the cytoplasm, inhibit apoptosis and enhance resistance of lens cells to stress (37,38). α-Crystallins, closely related to small HSPs, have been shown to act as molecular chaperones in other cell types (38,39). Lens alpha-, beta- and gamma-crystallins have been shown to be expressed in murine and human corneas (40), and members of each family have been shown to be upregulated by corneal degeneration in mice (41). RNA-seq analysis of the corneal epithelium of homozygous *Krt12*^hL132P/hL132P^ mice revealed that crystallins represented 10 of the most upregulated genes, suggesting a UPR in mutant corneal epithelial cells. Similarly upregulated, SerpinB2 has been shown to play a role in the aggregation of misfolded proteins into large protein inclusions that are thought to be less proteotoxic than smaller oligomeric complexes (42). In addition to their role in facilitating protein folding and assembly, chaperones have roles in the sorting of misfolded proteins to the proteasomal and lysosomal compartment for degradation and disposal in a process termed chaperone-assisted autophagy. Here, cytosolic material, such as protein aggregates, is encapsulated by a phagophore membrane to form an autophagosome which can fuse with the lysosome (43). Autophagy has been shown to modulate mutant keratin 18-containing inclusion formation (44).

Failure of the adaptive UPR to restore proteostasis and relieve ER stress in cells expressing mutant misfolded protein can overwhelm the proteosomal and lysosomal pathways and lead to activation of the apoptotic arm of the UPR (10). IHC demonstrated a strong staining of UPR markers CHOP and Caspase 12 in both mouse and human corneal tissues, with staining particularly pronounced in the corneal epithelium. Upregulation of CHOP and Caspase 12 results in increased apoptosis of the corneal epithelial cells in homozygous mice demonstrated by the number of TUNEL-positive cells. However, no increase in apoptosis was seen in the heterozygous mouse or the human MECD cornea. Although typically associated with an upregulation of apoptotic pathways, CHOP has also been found to play a protective role to improve protein-folding capacity and cell survival by transcriptional regulation of particular target genes and microRNAs such as *GADD34* (26) and miR-708 (45). Both *Grp78* and *Chop* are upregulated in a mouse model of Fuchs endothelial corneal dystrophy caused by dominant missense mutation of *Col8a2* (46), which results in endothelial cell apoptosis. Interestingly, lithium treatment upregulated expression of autophagy markers *P62*, *Tmem74*, *Tm9sf1* and *Tmem166* and improved endothelial cell survival, yet had no effect on the expression of either *Grp78* or *Chop*, and even increased expression of *Caspase 12*. This suggests that a balance of factors ultimately determines cell fate following UPR-induced CHOP expression. Although an alteration in expression of stress-related genes was not observed by RNA-seq analysis, here this may be due to an alteration in expression not large enough to be deemed significant with the small number of biological replicates used here. Although the proteomic analysis of the patient cornea did not determine an extensive list of all proteins found in the cornea, the most abundant proteins above the detection threshold are listed. Therefore, it is possible that stress proteins were differentially expressed in the human corneal samples, but unfortunately were expressed at a level below that quantifiable by the methods of detection used here.

Although there are many phenotypic similarities between human MECD and the mouse model, some differences do exist. A pronounced thickening of the corneal epithelium in the MECD mouse model was observed, which has not been described in human MECD. The thickening of Bowman's layer, observed in the human MECD tissue (5), is not found in this mouse model as the structure of the mouse cornea is somewhat different from that of human and lacks a Bowman's layer (47).

Expression of mutant Leu132Pro K12 results in differences in keratin protein profiles in the corneal epithelium between WT and mutant mice that largely recapitulate the changes found in the cornea of an MECD patient with the same mutation. Expression of keratins K6 and K16, which are known to be upregulated in response to stress (48), was observed. Although K6 is typically found in the upper layers of the corneal epithelium (49), K16 is usually absent but is upregulated during corneal epithelial wound healing where normal keratin expression is also dysregulated (50,51). Keratins K5 and K14 are expressed as a heterodimeric pair in the basal layer of the early prenatal cornea and corneal epithelium (52), with expression of K14 decreasing as K12 is increasingly expressed in more peripheral layers (53,54). In the absence of sufficient functional K12, K14 expression may be upregulated in compensation or as a consequence of this. These keratins appear to be coordinately regulated as downregulation of epithelial K12 expression seen in Pinin (*Pnn*)-null mice is observed together with ectopic expression of epidermal keratins K14 and K10 (also upregulated here) (55). Whereas K5 protein is found in all layers in the mouse cornea, its expression is decreased in both homozygous and heterozygous mutant mice. This contrasts with the upregulation of K5 observed in the human MECD cornea, and this difference may be attributed to the absence of an orthologue of the human *KRT3* gene in the mouse genome (56). Instead, in WT mice, K12 forms a cytoskeleton with K5 (7). Loss of functional K12 expression in the murine cornea correlates with decreased K5 expression, with a compensatory increase in K4 expression as previously observed in K5 null mice (7). In contrast, human differentiating limbal stem cells switch from K5/K14 to K3/K12 expression, with K5 expression not detectable in the central cornea (57,58). Thus, expression of mutant Leu132Pro keratin K12 in human and mouse corneas may be considered to result in an alteration of keratins consistent with an injured tissue attempting to compensate for loss of cytoskeletal integrity.

IHC revealed a downregulation in K12 expression in the human MECD cornea, whereas in contrast MS data showed an increase in K12, as quantified by the abundance of K12 peptides in the MECD cornea. Although K12 peptides may be increased, this is not a direct correlation to total protein, as K12 may be expressed and translated before readily undergoing proteolytic processing, thus reducing the ability of IHC to detect it. An increase in proteolysis in the MECD cornea would be expected in response to UPR (43) and would explain the observed reduction of K12 expression observed by IHC.

In addition, this research is indicative of the UPR having a role in the pathogenesis of MECD, which constitutes further evidence of the eye being overly susceptible to cellular stress-related disorders, as has been observed in numerous diseases including FECD (14–16), keratoconus (59–62), granular corneal dystrophy (63), cataracts (17) and diabetic retinopathy (64,65). However, the exact UPR pathway activated in MECD remains to be established. This apparently increased susceptibility to cellular stresses such as ER stress and oxidative stress could result from the high levels of UV absorption in the eye (66,67), a known stress-inducing agent that additionally stimulates protein misfolding and DNA damage (68). Further research discerning the exact role of these UPR components in the MECD cornea and the potential role of UV light in this disorder is still required before a definitive pathomechanism can be elucidated. Although the phenotype observed in the homozygous mouse is more severe than that in the heterozygous mouse, similar phenotypic changes in corneal structure, expression of additional keratins and altered expression of Caspase 12 are apparent in both. This is similar to other previously reported corneal mouse models, such as Fuchs, in which a homozygous mouse is used as a model for the autosomal dominant, heterozygous human disease (15). This mouse model will serve as a test-bed for therapies targeted against corneal stress responses and, additionally, for other keratinopathies caused by similar mutations.

## Materials and Methods

### Design and generation of a transgenic mouse expressing mutant humanized K12 (L132P)

The targeting vector and mouse were manufactured by Taconic Artemis (Köln, Germany). The targeting strategy is shown in Figure 1. The full human *KRT12* gene including introns, 5′- and 3′-UTRs and a 247 bp sequence downstream of the *KRT12* 3′-UTR was cloned and the following modifications made: the L132P mutation was introduced in exon 1; an FRT-flanked neomycin resistance gene (Neo^R^) was inserted into intron 2; an F3-flanked puromycin resistance gene (Puro^R^) was inserted into intron 7; a FLAG-HA coding sequence (21) was placed immediately before the termination codon and an MTC (Supplementary Material, Fig. S1) was inserted within the 3′-UTR of the endogenous *KRT12* gene sequence. The resulting *KRT12* transgene was inserted in a mouse targeting vector with a 4.1 kb 5′ short homology arm (SHA) and a 6.2 kb 3′ long homology arm (LHA), corresponding to the regions flanking the mouse *Krt12* gene (Fig. 1). The MTC contained ∼40 nt including and surrounding the *KRT3* mutations c.1525G>A (p.Glu509Lys, E509K), c.1508G>C (p.Arg503Pro, R503P) and c.1493A>T (p.Glu498Val, E498V) and the *KRT12* mutations c.395T>C (p.Leu132Pro, L132P) and c.404G>C (p.Arg135Thr, R135T). Regions were arranged sequentially as shown in Supplementary Material, Figure S1, with flanking 5′ *Nhe*I and 3′ *Xho*I restriction sites included. Mice were bred to homozygosity with heterozygote/heterozygote matings to achieve age-matched littermates with all three genotypes. The various genotypes are referred to as WT = Krt12^+/+^ (also +/+), heterozygote = Krt12^+/hL132P^ (also +/−) and homozygote = Krt12^hL132P/hL132P^ (also −/−).

### Mouse genotyping

Genomic DNA was extracted from ear biopsies by alkaline lysis (69). Primers were designed enabling incorporation of a single PCR three-primer multiplex with one common primer. Genotyping was achieved using a common forward primer hK12HU.G.1F (5′-CGA GAG TTG GAA CGC AGA GA-3′) in equimolar amounts to primers hK12HU.G.1R (5′-TCC CTA TCC CCA TTC CTC CC-3′) and hK12HU.G.2R (5′-CCT GGG TCT GCC TAT CAC AC-3′), the former detecting the *Krt12* allele with a 404 bp product and the latter the *KRT12* allele with a 201 bp product (Supplementary Material, Fig. S4). The PCR temperature cycle was as follows: 95°C for 3 min, 35 cycles of 95°C for 15 s, 57°C for 15 s, 72°C for 30 s followed by 5 min at 72°C.

### *In vivo* slit-lamp evaluation of humanized MECD mouse cornea

Slit-lamp assessment was carried out on mice at 16 weeks (*n* = 6 per genotype). They were assessed using a portable digital slit lamp (Hawkeye, Dioptrix, Toulouse, France), with images taken using a Pentax Optio S60 camera. Both eyes of each mouse were inspected and imaged. Mouse eyes were also stained with fluorescein (Minims Fluorescein Sodium 2% w/v; Bausch & Lomb, Surrey, UK) prior to assessment and images were taken. Eyes were kept hydrated with Balanced Sterile Saline Solution (Beaver-Visitec International, Abington, UK) during assessment.

### Electron microscopy of humanized MECD mouse cornea

For SEM, the corneal surface was evaluated on one mouse per genotype at age 7 weeks. Mouse eyes were enucleated, fixed, processed and imaged, as described by Fu and colleagues (70).

For TEM of corneal sections, whole mouse eyes were enucleated and immediately immersed in fixative [2.5% glutaraldehyde and 4% paraformaldehyde (PFA) in 0.1 m sodium cacodylate buffer, pH 7.3], with the back of the eye removed after 15 min to aid fixation. Following fixation for 48 h, the corneas were dehydrated through an ethanol gradient and propylene oxide (71) and embedded in Durcupan resin (Sigma, -Aldrich, Dorset, UK). Ultra-thin sections (70 nm) were cut, processed and imaged as described previously (18) with preliminary observations made, followed by confirmation by two blinded individuals.

### Preparation of mouse and human tissue

Mice were euthanized by CO_2_ asphyxiation. For extraction of protein or RNA, mouse eyes were immediately enucleated, full thickness corneas dissected as required and placed in 1.5 ml tubes before being snap-frozen in liquid nitrogen and stored at −80°C. For histology, whole eyes were enucleated, placed in histology cassettes (VWR, Lutterworth, UK) and immediately submersed for 24 h in Davidson's fixative with final concentrations of 36% ethanol, 12.5% glacial acetic acid and 25% neutral-buffered formalin made up in distilled water (72).

Corneal tissue from a 44-year-old female MECD patient, and known to be heterozygous for the KRT12-Leu132Pro mutation through previous genotyping (20), was collected after surgery and separated into two pieces. One portion of the tissue, consisting of all corneal layers, was immediately snap-frozen at −80°C for proteomic analysis by MS. Control tissue consisting of all corneal layers was collected postmortem and processed in the same manner. The second portion was fixed in 4% PFA for histological examination. Healthy corneal tissue acquired from the Manchester Eye Bank was processed in the same way and used as control tissue.

### Immunohistochemistry

After fixation, eyes were dehydrated through graded alcohol solutions and xylene prior to embedding in paraffin wax. Sections of 7 μm were cut and placed on 3-aminopropyltriethoxysilane-coated glass slides and incubated overnight at 37°C. Slides were then stored at room temperature until use.

For mouse tissue, IHC was performed using the protocol outlined by Leachman *et al.* (73), with the following modifications; all washes were in TBST (TBS containing 0.5% Tween-20) and endogenous peroxidases were blocked with 3% hydrogen peroxide made up in phosphate-buffered saline (PBS). Immediately prior to primary antibody incubation, tissues were blocked for 20 min with 10% serum in PBS. The serum used was from the same host species in which the secondary antibodies were raised. Mouse sections were stained for HA (1:200; C29F4, Cell Signaling Technology, Hitchin, UK), K5 (1:800; a gift from E.B. Lane, A-Star Institute, Singapore), K14 (1:400; LL001, Santa Cruz, Biotechnology, Heidelberg, Germany (70)), K6 [1:500; a gift from Pierre Coulombe (74)], K16 [1:500; a gift from Pierre Coulombe (75)], Caspase 12 (1:200; ab62484, Abcam, Cambridge, UK) or CHOP (DDIT3) (1:100; ab179823, Abcam). A horseradish peroxidase (HRP) conjugated secondary antibody was used to allow visualization of HA, K5, K14, K6 and K16 proteins. A fluorescein isothiocyanate (FITC)-conjugated goat anti-rabbit polyclonal antibody (Dako, Glostrup, Denmark) at a dilution of 1:1000 was used to visualize Caspase 12 and CHOP. These slides were mounted in Ultra-Cruz mounting medium (Santa Cruz, Heidelberg, Germany) before being imaged by standard immunofluorescent microscopy. Haematoxylin and eosin (H&E) staining was performed following standard protocols.

For human tissue, following rehydration, antigen retrieval was achieved by proteinase K (Fisher Scientific, Loughborough, Uk) (10 μg/ml final concentration) at 37°C for 45 min. Non-specific antibody binding was blocked by incubation in 5% goat serum, made up in PBS, for 1 h. Tissue was stained for K12 (1:100), K5 (1:1000), K14 (1:100), K6 (1:200), K16 (1:200), Caspase 12 (1:200) or CHOP (1:100). All antibody dilutions were made up in 2% goat serum in PBS. After incubation overnight at 4°C, slides were washed in PBS, three times for 15 min each, and incubated for 1 h at room temperature with FITC-conjugated goat anti-rabbit polyclonal antibody (Dako, Glostrup, Denmark) at a dilution of 1:1000 and washed in PBS as before. Slides were mounted in Ultra-Cruz mounting medium (Santa Cruz, Biotechnology, Heidelberg, Germany) before being imaged by immunofluorescent microscopy.

### Preparation of corneal lysates for protein analysis

Lysis buffers A and B were prepared as follows and used in a 1:1 ratio for protein lysis and gel loading. Buffer A consisted of an aqueous solution containing 1 m unbuffered TRIS base and 13% sodium dodecyl sulphate (SDS). Buffer B consisted of 0.2 m DTT, 30% glycerol and 0.002% bromophenol blue to which HCl was added until the colour changed from blue to orange. Both buffers were stored at −20°C until use. Frozen corneas were crushed with a sterile plastic dounce in 100 µl of premixed in-house lysis buffers A and B. Samples were incubated at 70°C for 10 min and sonicated for a total of 30 s at 30% amplification applied in 10 s pulses. Cell debris was pelleted at 16 000*g* for 15 min at 4°C and the supernatant removed to a fresh tube.

### Western blotting

Mouse tissue protein analysis was performed with 5 µl of total protein lysate per lane. Proteins were resolved on 4–12% BIS/Tris gels (Life Technologies) on an XCell II (Life Technologies) system and MOPS-SDS buffer (Life Technologies, Paisley, UK) and assessed for protein loading by Coomassie blue staining. Subsequent gel loading volume was normalized on the basis of total protein and β-actin intensity. Western blotting analysis was performed as described previously (76) with primary antibodies for K12 (1:1000; Moravian Biotech, Brno, Czech Republic), HA (1:1000) and β-actin (1:5000; A2228, Sigma).

Western blotting of protein lysates for K5 (1:5000), K14 (1:5000), K6 (1:2000) and K16 (1:2500) was performed using the same procedure, but a HRP-conjugated secondary antibody was used and membranes imaged following the manufacturer's instructions.

### RNA-seq analysis

RNA from frozen corneas of 7–9-week-old mice from each genotype (*n* = 6 individuals for RNA-seq and *n* = 3 pooled for qPCR) was extracted after homogenization and lysis using the RNeasy Plus Minikit together with an on-column DNase digestion (Qiagen Manchester, UK).

RNA from single corneas (*n* = 6 per genotype) was sequenced by the Genomic Sequencing Unit (University of Dundee, UK). Libraries were constructed using the TruSeqRNA v2 protocol following the manufacturer's instructions (Illumina, Saffron Walden, UK) and were normalized to 10 nm and pooled in three sets of six. Each pool contained two WT samples, two heterozygote samples and two homozygote samples. Each sample pool was sequenced using a HiSeq2000 (Illumina) as paired-end 100 bp reads.

For RNA-seq data analysis, reads were aligned with STAR (version 2.3.0e_r291) against the mouse reference genome (Ensembl release 74), with the following parameters: outFilterMultimapNmax 2—outFilterMismatchNmax 5—outFilterType BySJout—outSJfilterIntronMaxVsReadN 5000 10 000 15 000 20 000. Approximately 87% of reads were found to align. Aligned reads were annotated to genes with htseq-count (version 0.5.4p5) in ‘union’ mode, referenced to Ensembl (release 74) gene annotations. The resulting gene read counts were used to determine differentially expressed genes. All 18 samples were imported in R (v. 3.0.2), and any gene with fewer than 36 reads (i.e. two per sample) in total was excluded from further analysis (leaving 16 845 genes). edgeR (v. 3.4.0) was used in GLM mode across all samples to identify all differentially expressed genes with false discovery rate-adjusted *P* < 0.05 in the WT versus heterozygous and WT versus homozygous comparisons.

### qRT-PCR

An equimolar quantity of VIC-labelled mGAPDH primers and probes (Applied Biosystems, assay no. 4352339E) was added as an internal control into each keratin qPCR. RNA was reverse-transcribed according to the manufacturer's protocol (High Capacity Reverse Transcription kit, Life Technologies). *Krt12/KRT12* qPCR reactions (perfeCTa^®^ qPCR toughmix; Quanta Biosciences, Gaithersburg, MD, USA) contained cDNA equivalent to 1.25 ng/µl total RNA. qPCR reactions for other keratins (TaqMan Universal Mastermix II, Life Technologies) contained cDNA equivalent to 1.8 ng/µl. A TaqMan 7900HT machine (Applied Biosystems Paisley, UK) was used for quantification of triplicate samples using the ΔΔCt method (77).

### TUNEL assay and quantification

A TUNEL assay to identify apoptotic cells was performed on sections containing ocular tissue from WT, heterozygous or homozygous MECD mice. After deparaffinization, rehydration and antigen retrieval, as described previously, apoptotic cells were stained using the *In Situ* Cell Death Detection kit (Fluorescein; Roche, Burgess Hill, Surrey, UK) following the manufacturer's instructions. Slides were mounted and imaged using standard fluorescent microscopy. Corneas from three mice of each genotype were sectioned, and three images were taken and quantified for each cornea. The numbers of live and apoptotic cells were determined from images taken from the central corneas. The proportion of apoptotic cells was calculated for each section by dividing the number of apoptotic cells by DAPI-stained cells.

### Sample preparation for LC-MS/MS—in-solution digest

Patient and control tissues were lyophilized for 2 h using a vacuum concentrator and ground into powder under liquid nitrogen using a pestle and motor. In total, 1.2 mg of each tissue was treated with 0.66 m CNBr in 70% trifluoroacetic acid overnight at 23°C. The samples were lyophilized and resuspended in 6 m urea in 0.2 m Tris–HCl, pH 8.3, before being reduced in 5 mm dithiothreitol (DTT) for 30 min and then alkylated in 15 mm iodoacetamide for 30 min. Samples were digested with Lys-C protease (Sigma, -Aldrich) 1:1000 w/w for 6 h at 37°C and then diluted four times using 0.1 m Tris–HCl, pH 8.3, followed by a second digestion step overnight with 1:50 w/w sequencing grade-modified trypsin (Sigma, -Aldrich) at 37°C for 16 h. The samples were desalted using POROS 50 R2 RP column material (Applied Biosystems) packed in GELoader Tips (Eppendorf, Stevenage, UK).

### Liquid chromatography (LC)-MS/MS analysis

LC-MS/MS analyses were performed on an EASY-nLC II system (Thermo Fisher Scientific, Paisley, UK) connected to a TripleTOF 5600 + mass spectrometer (AB SCIEX, Copenhagen, Denmark) equipped with a NanoSpray III source (AB SCIEX) and operated under Analyst TF 1.6.0 control. The CNBr and trypsin-cleaved samples were dissolved in 0.1% formic acid, injected and trapped on an in-house packed trap column (2 cm × 100 μm i.d.) using RP ReproSil-Pur C18-AQ 3 μm resin (Dr Maisch GmbH, Ammerbuch-Entringen, Germany). Peptides were eluted from the trap column and separated on a 15 cm analytical column (75 μm i.d.), pulled and packed in-house with RP ReproSil-Pur C18-AQ 3 μm resin (Dr Maisch GmbH) and sprayed directly into the mass spectrometer. Peptides were eluted at a flow rate of 250 nl/min using a 50 min gradient from 5 to 35% phase B (0.1% formic acid and 90% acetonitrile). The acquisition method used for the extracted ion chromatogram (XIC) quantification was set up as an information-dependent acquisition experiment collecting up to 25 MS/MS spectra in each 1.6 s cycle using an exclusion window of 12 s. The Meesmann and control corneal samples were each run in triplicate on the mass spectrometer.

### Protein quantitation

Raw data from the LC-MS/MS were converted to mgf format using AB SCIEX MS Data Converter beta 1.1 and the ‘proteinpilot mgf’ parameters, and peptides were searched using an in-house Mascot search engine (Matrix Science, London, UK; version: 2.5.0) and queried against the Swiss-Prot database (version: 2015_09). CNBr + trypsin was selected as the digestion enzyme allowing one missed cleavage. Carbamidomethyl was entered as a fixed modification, whereas hydrozylation of proline, oxidation of methionine and C-terminal conversion of methionine to homoserine and homoserine lactone by CNBr were used as variable modifications. The data were searched with a mass tolerance of the precursor and product ions of 10 ppm and 0.2 Da using ESI-QUAD-TOF as the instrument setting. All searches had a *P* < 0.01 applied and an expected value of 0.005. Only protein hits identified by a minimum of two peptides with Mascot ion scores above 30 were considered for further analyses. In addition, porcine tryptic peptides identical to human peptides were removed as porcine trypsin was used during the processing of the samples.

All data were imported and processed in MS Data Miner v. 1.3.0 (78). Proteins were arranged on the basis of their exponentially modified Protein Abundance Index values (emPAI). Relative abundance was termed as the emPAI value for each protein in the sample divided by the sum of the emPAI values of all proteins present within the tissue specimens.

### Statistics

All error bars indicate the standard error of the mean across samples of the same biological significance. A one-way analysis of variance was performed to calculate significance in the differences in keratin protein expression in mouse corneas, assessed by immunoblotting. In all other cases, a Student's *t*-test was used to calculate significance; *P* < 0.05 was deemed to be significant (*P < 0.05, **P < 0.01 and ***P < 0.001).

### Study approval

For all human tissues, ethical issues were handled according to Danish healthcare law and in accordance with the local Ethics Committee and national health authorities. All work was carried out in accordance with the Declaration of Helsinki. Informed consent was received from participants prior to inclusion in this study.

## Supplementary Material

Supplementary Material is available at *HMG* online.

## Funding

This work was supported by the United Kingdom Medical Research Council (MRC) grants G0801742 and G0802780 (to W.H.I.M), Fight for Sight (UK) grants 1880 and 1450/1451 (to W.H.I.M. and C.B.T.M.), Wellcome Trust Strategic Award 098439/Z/12/Z (to W.H.I.M.) and the Danish Council for Independent Research-Medical Sciences (DFF-4004-00471). Funding to pay the Open Access publication charges for this article was provided by The Wellcome Trust.

## Supplementary Material

Supplementary Data
