# Supplementary material for: Keratin 12 missense mutation induces the unfolded protein response and apoptosis in Meesmann epithelial corneal dystrophy
Source: Hum Mol Genet. 2016 Jan 11;25(6):1176–91. doi: 10.1093/hmg/ddw001 (PMC4764196; doi:10.1093/hmg/ddw001)
Supplement: Supplementary Data [file supp_25_6_1176__index.html]

Keratin 12 missense mutation induces the unfolded protein response and apoptosis in Meesmann epithelial corneal dystrophy — Keratin 12 missense mutation induces the unfolded protein response and apoptosis in Meesmann epithelial corneal dystrophy — Supplementary Data 

# Keratin 12 missense mutation induces the unfolded protein response and apoptosis in Meesmann epithelial corneal dystrophy

## Supplementary Data

Supplementary Data

- Supplementary Figures - docx file
- Supplementary Tables - docx file
