# Supplementary material for: Keratin 12 missense mutation induces the unfolded protein response and apoptosis in Meesmann epithelial corneal dystrophy
Source: Hum Mol Genet. 2016 Jan 11;25(6):1176–91. doi: 10.1093/hmg/ddw001 (PMC4764196; doi:10.1093/hmg/ddw001)
Supplement: Supplementary Data [file supp_ddw001_ddw001supp_figs.docx]

**
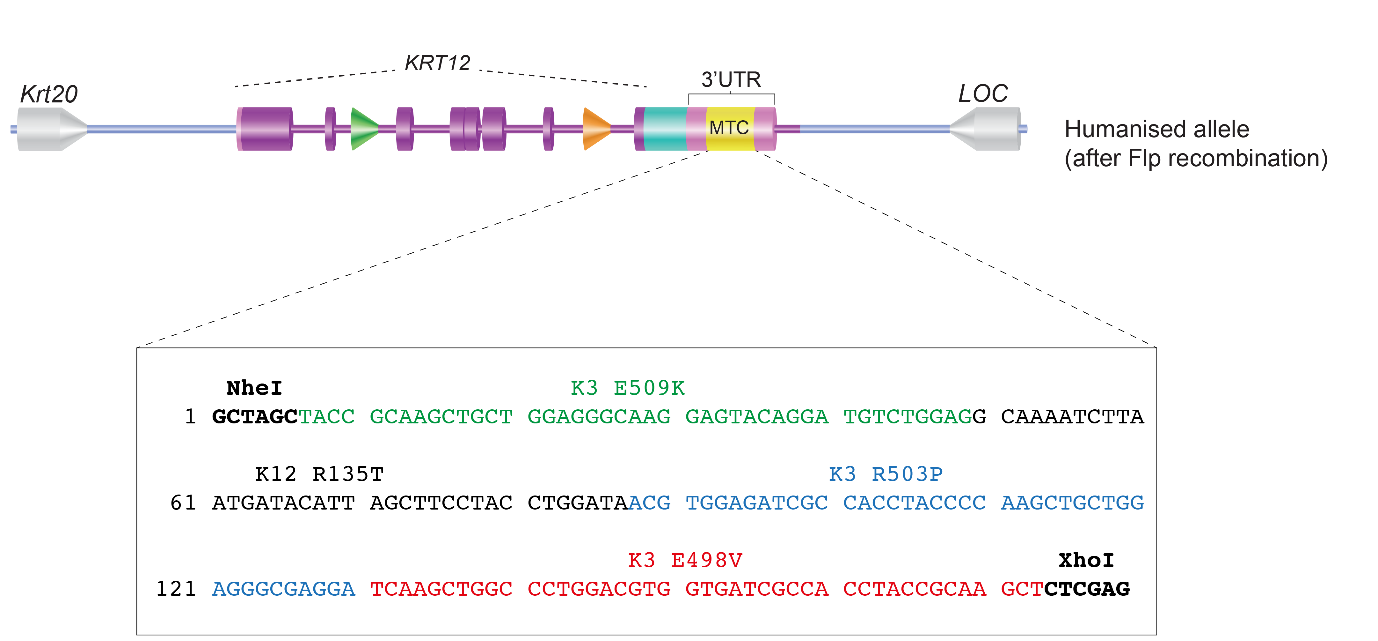
Supplementary Figure 1 – Transgene multi-target cassette**

The target sequences against the K3 mutations E509K, R503P and E498V and K12 mutations L132P and R135T were arranged in a MTC as illustrated.

**
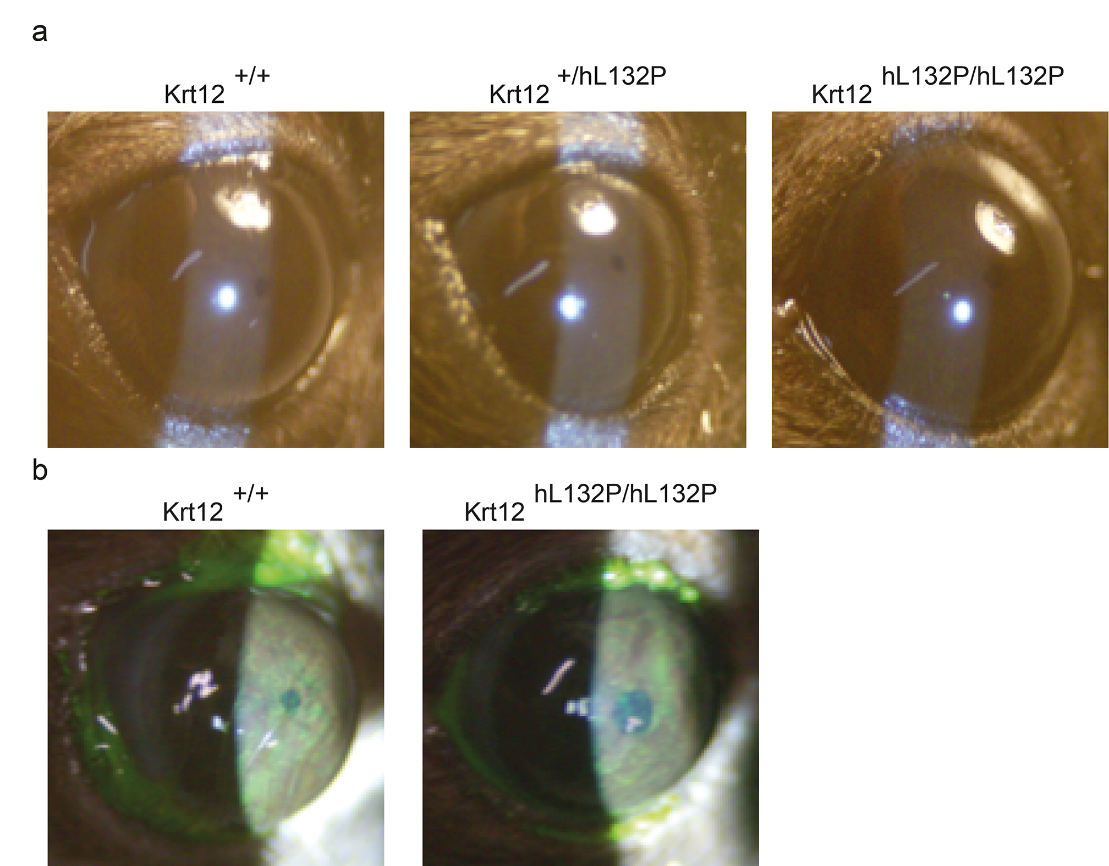
Supplementary Figure 2 - Slit-lamp examination of corneas for gross phenotypic changes**

(A) Mice (n=6 per genotype) were examined at 8, 12 and 16 wks old by slit lamp to look for overt changes in corneal clarity structure typical of the MECD phenotype; however, no differences were observed. (B) At 16 wks the eyes were also stained with fluorescein to highlight any changes to the corneal surface. Again no significant differences were observed across the genotypes.

**
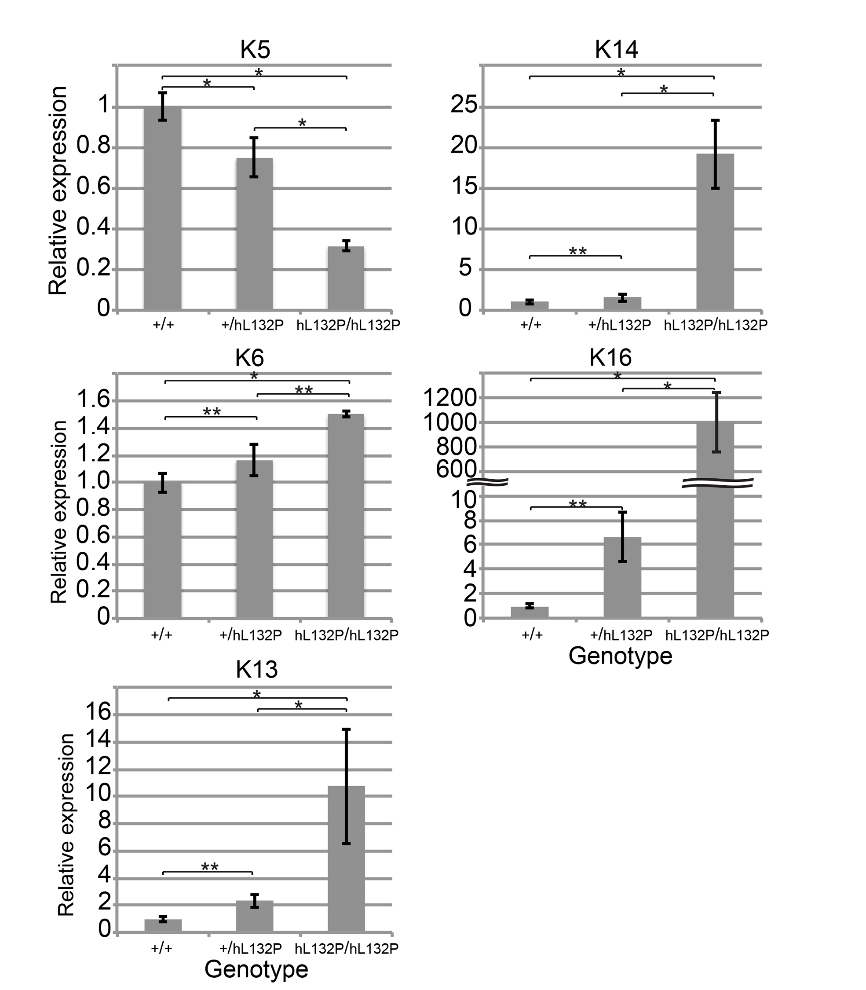
**

**Supplementary Figure 3 – Quantification of keratin immunoblotting for MECD mouse corneas**

Immunoblotting from Figure 4C of corneal lysates, (n=3 per genotype) for keratins K5, K14, K6, K16 and K13, underwent semi-quantification by densitometry, with expression normalised to that of β-actin. A significant decrease was observed in K5 expression between both heterozygous and homozygous genotypes when compared to wild type mice (p<0.01, p<0.01). The protein expression of the 4 other keratins is significantly increased with increasing levels of transgene expression in both the heterozygous (all p<0.01) and homozygous (all p<0.05) mice.

**
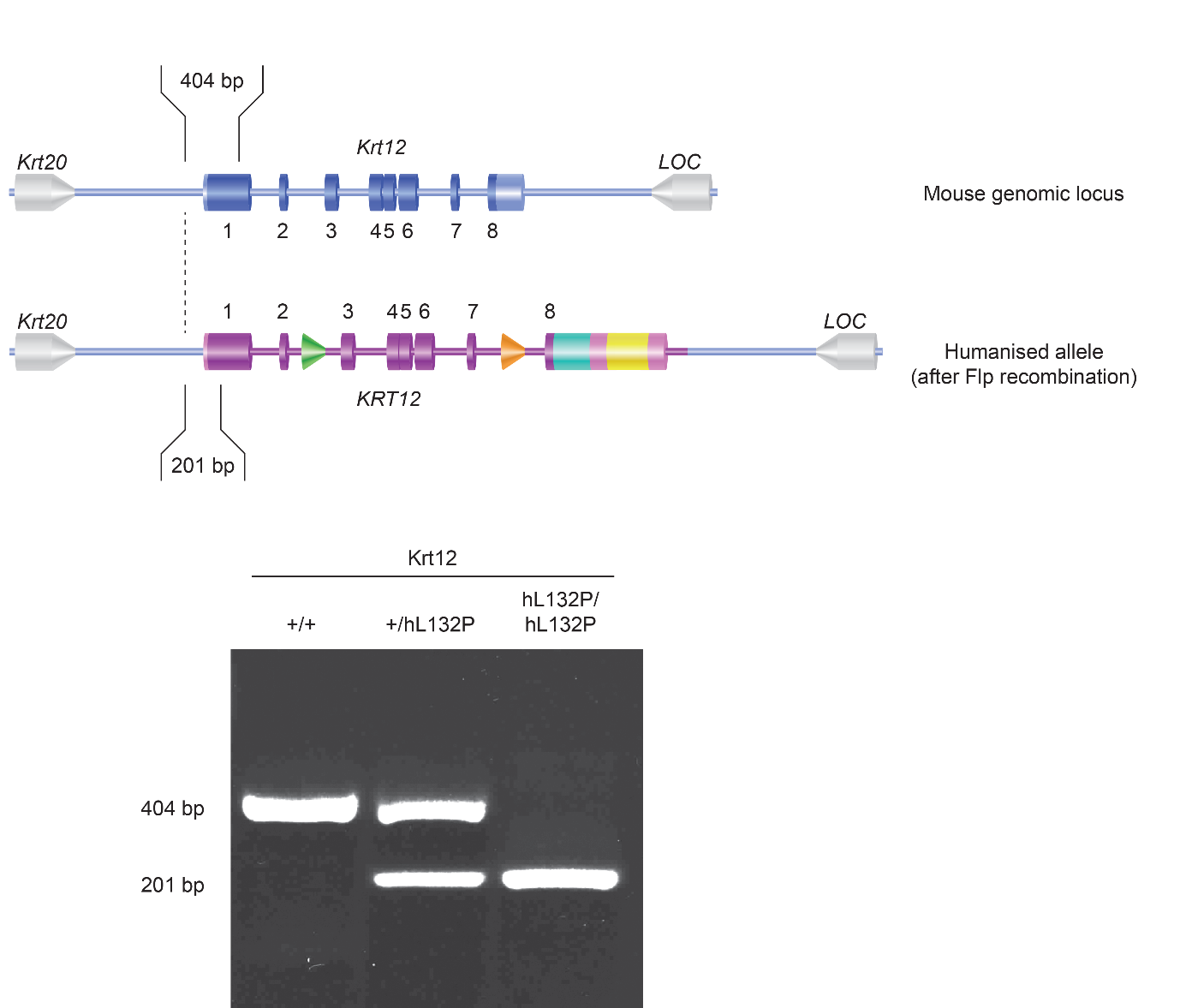
Supplementary Figure 4 - Genotyping strategies for Krt12^hL132P^mouse**

(A) The gene structure of the WT and humanised *Krt12* alleles and the positions of the allele-specific PCR primer sets used for genotyping are shown. PCR products shown in black are the products from the original PCR design based on primers and protocols from the mouse supplier Taconic Artemis. A modified single three-primer multiplex PCR design was subsequently used to streamline the genotyping process (pink font). (B) Genotyping results were determined from the original two-reaction PCR design. Both reactions contained additional primers to detect the CD79b gene as an internal control; a 196 bp product detects the WT allele (left panel) and a 352 bp product detects the humanised allele (right panel). (C) Results from the multiplexed PCR with 404 and 201 bp products detecting the WT and humanised alleles respectively.
