# Supplementary material for: Keratin 12 missense mutation induces the unfolded protein response and apoptosis in Meesmann epithelial corneal dystrophy
Source: Hum Mol Genet. 2016 Jan 11;25(6):1176–91. doi: 10.1093/hmg/ddw001 (PMC4764196; doi:10.1093/hmg/ddw001)
Supplement: Supplementary Data [file supp_ddw001_ddw001supp_tables.docx]

**Supplementary Table S1. Highly dysregulated genes in the corneas of heterozygous and homozygous Krt12^hL132P^ mice**

| **Table S1. Highly dysregulated genes in the corneas of heterozygous and homozygous Krt12^hL132P^ mice** | | | | | |
| --- | --- | --- | --- | --- | --- |
|  |  | Gene | Log_2_ (Fold Change) | Fold Change | P value, FDR adjusted |
|  |  |  |  |  |  |
|  |  |  |  |  |  |
| WT vs. heterozygote | upregulated | *Malat1* | 0.595 | 1.5 | 5.40E-04 |
|  | downregulated | *Ltf* | -0.813 | 0.57 | 0.026 |
|  |  |  |  |  |  |
| WT vs. homozygote | upregulated | *Crybb2* | 9.032 | 523.5 | 5.90E-05 |
|  |  | *Cryba1* | 8.222 | 298.6 | 6.80E-04 |
|  |  | *Saa3* | 7.601 | 194.1 | 2.50E-12 |
|  |  | *Crygb* | 7.261 | 153.4 | 0.0019 |
|  |  | *Cryba2* | 7.251 | 152.4 | 4.10E-04 |
|  |  | *Crygc* | 6.864 | 116.5 | 9.10E-04 |
|  |  | *Cryaa* | 6.434 | 86.4 | 3.70E-06 |
|  |  | *Crygd* | 5.911 | 60.2 | 0.0019 |
|  |  | *Sprr1b* | 5.601 | 48.5 | 0.0036 |
|  |  | *Crybb1* | 5.491 | 45 | 0.0038 |
|  |  | *Crygs* | 5.409 | 42.5 | 4.30E-04 |
|  |  | *Serpinb2* | 4.541 | 23.3 | 6.00E-04 |
|  |  | *Dynap* | 4.449 | 21.8 | 7.20E-04 |
|  |  | *Krt16* | 4.413 | 21.3 | 8.30E-11 |
|  |  | *Ccl5* | 4.028 | 16.3 | 1.60E-04 |
|  |  | *Chi3l1* | 3.675 | 12.8 | 2.90E-08 |
|  |  | *Ifi205* | 3.28 | 9.7 | 0.004 |
|  |  | *Crybb3* | 3.07 | 8.4 | 0.0022 |
|  |  | *Sh2d5* | 2.985 | 7.9 | 3.30E-05 |
|  |  | *Mmp3* | 2.956 | 7.8 | 0.0095 |
|  |  | *Malat1** | 0.533 | 1.4 | 4.40E-05 |
|  | downregulated | *Ltf** | -0.525 | 0.7 | 0.035 |
|  |  | *Gm10664* | -3.544 | 0.09 | 3.40E-18 |
|  |  | *Chrnb3* | -4.069 | 0.06 | 4.10E-20 |
|  |  | *A830036E02Rik* | -5.317 | 0.03 | 1.40E-94 |

**Supplementary Table S2. Relative amounts of the most abundant proteins in control & MECD tissue**

| **Table S2. Relative amounts of the most abundant proteins in control & MECD tissue** | | | | | |
| --- | --- | --- | --- | --- | --- |
| **Control tissue** | | | **MECD Tissue** | | |
|  | **Name** | **Amount (%)** |  | **Name** | **Amount (%)** |
| 1 | Serum albumin | 12.28 | 1 | Collagen alpha-1(I) chain | 9.50 |
| 2 | Collagen alpha-1(I) chain | 10.72 | 2 | Serum albumin | 9.07 |
| 3 | Collagen alpha-2(I) chain | 5.32 | 3 | Collagen alpha-2(I) chain | 4.06 |
| 4 | Ig kappa chain C region | 5.06 | 4 | Ig kappa chain C region | 3.48 |
| 5 | TGFBI | 4.28 | 5 | TGFBI | 2.86 |
| 6 | Keratocan | 3.23 | 6 | Histone H4 | 2.64 |
| 7 | Vimentin | 2.53 | 7 | Keratin, type II cytoskeletal 5 | 2.61 |
| 8 | Decorin | 2.35 | 8 | Keratocan | 2.41 |
| 9 | Ig lambda-2 chain C regions | 2.33 | 9 | Ig lambda-2 chain C regions | 2.41 |
| 10 | Collagen alpha-3(VI) chain | 2.06 | 10 | Histone H2B type 1-B | 2.15 |
| 11 | Lumican | 2.02 | 11 | Keratin, type I cytoskeletal 14 | 2.10 |
| 12 | Aldehyde dehydrogenase, dimeric NADP-preferring | 1.86 | 12 | Lumican | 2.06 |
| 13 | Mimecan | 1.85 | 13 | Histone H2B type 1-C/E/F/G/I | 1.93 |
| 14 | Ig gamma-1 chain C region | 1.83 | 14 | Keratin, type II cytoskeletal 6A | 1.92 |
| 15 | Histone H2B type 1-H | 1.68 | 15 | Collagen alpha-3(VI) chain | 1.80 |
| 16 | Histone H2B type 1-C/E/F/G/I | 1.66 | 16 | Decorin | 1.76 |
| 17 | Serotransferrin | 1.38 | 17 | Apolipoprotein A-I | 1.73 |
| 18 | Apolipoprotein A-I | 1.25 | 18 | Hemoglobin subunit beta | 1.55 |
| 19 | Histone H4 | 1.23 | 19 | Ig gamma-1 chain C region | 1.52 |
| 20 | Apolipoprotein A-II | 1.22 | 20 | Aldehyde dehydrogenase, dimeric NADP-preferring | 1.40 |
| 21 | Collagen alpha-1(VI) chain | 1.14 | 21 | Keratin, type I cytoskeletal 13 | 1.37 |
| 22 | Prolargin | 1.13 | 22 | Apolipoprotein A-II | 1.34 |
| 23 | Ig kappa chain V-III region SIE | 1.04 | 23 | Collagen alpha-1(VI) chain | 1.28 |
| 24 | Annexin A2 | 0.96 | 24 | Keratin, type I cytoskeletal 12 | 1.27 |
| 25 | Ig gamma-3 chain C region | 0.95 | 25 | Vimentin | 1.17 |
| 26 | Collagen alpha-2(VI) chain | 0.93 | 26 | Mimecan | 1.13 |
| 27 | Ig gamma-2 chain C region | 0.91 | 27 | Biglycan | 0.98 |
| 28 | Alpha-enolase | 0.80 | 28 | Actin, cytoplasmic 1 | 0.98 |
| 29 | Ig alpha-1 chain C region | 0.79 | 29 | Keratin, type I cytoskeletal 19 | 0.92 |
| 30 | Collagen alpha-2(V) chain | 0.77 | 30 | Annexin A2 | 0.88 |
| 31 | Immunoglobulin lambda-like polypeptide 5 | 0.76 | 31 | Collagen alpha-2(VI) chain | 0.83 |
| 32 | Biglycan | 0.75 | 32 | Alpha-enolase | 0.83 |
| 33 | Histone H2A type 1-B/E | 0.74 | 33 | Hemoglobin subunit alpha | 0.80 |
| 34 | Ig kappa chain V-III region GOL | 0.74 | 34 | Ig gamma-3 chain C region | 0.79 |
| 35 | Alpha-1-antitrypsin | 0.73 | 35 | Serotransferrin | 0.78 |
| 36 | Pigment epithelium-derived factor | 0.71 | 36 | Alpha-1-antitrypsin | 0.76 |
| 37 | Ig heavy chain V-III region TEI | 0.67 | 37 | L-lactate dehydrogenase A chain | 0.68 |
| 38 | Collagen alpha-1(XII) chain | 0.66 | 38 | Pigment epithelium-derived factor | 0.68 |
| 39 | Ig heavy chain V-III region BRO | 0.64 | 39 | Immunoglobulin lambda-like polypeptide 5 | 0.67 |
| 40 | Protein S100-A6 | 0.64 | 40 | Prolargin | 0.65 |
| 41 | Ig heavy chain V-III region CAM | 0.61 | 41 | Ig kappa chain V-III region SIE | 0.65 |
| 42 | Dermatopontin | 0.57 | 42 | Ig gamma-2 chain C region | 0.64 |
| 43 | L-lactate dehydrogenase A chain | 0.57 | 43 | Apolipoprotein D | 0.60 |
| 44 | Complement C3 | 0.56 | 44 | Collagen alpha-2(V) chain | 0.60 |
| 45 | Keratin, type II cytoskeletal 5 | 0.53 | 45 | Dermatopontin | 0.56 |
| 46 | Histone H3.1t | 0.52 | 46 | Ig alpha-1 chain C region | 0.56 |
| 47 | Alpha-1-acid glycoprotein 1 | 0.51 | 47 | Histone H2A type 1-B/E | 0.52 |
| 48 | Apolipoprotein D | 0.51 | 48 | Alpha-1-antichymotrypsin | 0.49 |
| 49 | Collagen alpha-1(V) chain | 0.45 | 49 | Histone H3.1t | 0.46 |
| 50 | Collagen alpha-1(III) chain | 0.44 | 50 | Alpha-1-acid glycoprotein 1 | 0.45 |
| 51 | Peptidyl-prolyl cis-trans isomerase A | 0.43 | 51 | Collagen alpha-1(III) chain | 0.43 |
| 52 | Transthyretin | 0.41 | 52 | Peroxiredoxin-1 | 0.42 |
| 53 | Alpha-1-antichymotrypsin | 0.38 | 53 | Keratin, type II cytoskeletal 3 | 0.42 |
| 54 | MAM domain-containing protein 2 | 0.36 | 54 | Prolactin-inducible protein | 0.41 |
| 55 | Annexin A5 | 0.31 | 55 | Prostaglandin-H2 D-isomerase | 0.41 |
| 56 | Ig kappa chain V-I region Mev | 0.31 | 56 | Heat shock protein beta-1 | 0.41 |
| 57 | Ig lambda chain V-I region HA | 0.31 | 57 | Peptidyl-prolyl cis-trans isomerase A | 0.38 |
| 58 | Ig lambda chain V-III region LOI | 0.31 | 58 | 14-3-3 protein sigma | 0.37 |
| 59 | Clusterin | 0.31 | 59 | Transthyretin | 0.36 |
| 60 | Prostaglandin-H2 D-isomerase | 0.29 | 60 | Elongation factor 1-alpha 1 | 0.34 |
| 61 | Ig kappa chain V-III region VG | 0.29 | 61 | Collagen alpha-1(V) chain | 0.34 |
| 62 | Annexin A1 | 0.29 | 62 | Protein S100-A6 | 0.32 |
| 63 | Ig kappa chain V-II region Cum | 0.29 | 63 | Protein S100-A8 | 0.30 |
| 64 | Ig heavy chain V-III region VH26 | 0.29 | 64 | Complement C3 | 0.28 |
| 65 | Tetranectin | 0.28 | 65 | Ig kappa chain V-I region DEE | 0.27 |
| 66 | Gelsolin | 0.24 | 66 | Ig kappa chain V-I region Wes | 0.27 |
| 67 | Haptoglobin | 0.24 | 67 | C-type lectin domain family 11 member A | 0.27 |
| 68 | Histone H3.3C | 0.23 | 68 | Ig kappa chain V-I region EU | 0.27 |
| 69 | Thrombospondin-4 | 0.22 | 69 | Ig lambda chain V-III region LOI | 0.27 |
| 70 | Collagen alpha-2(VIII) chain | 0.21 | 70 | Ubiquitin-40S ribosomal protein S27a | 0.27 |
| 71 | Serum amyloid P-component | 0.21 | 71 | Clusterin | 0.26 |
| 72 | Angiopoietin-related protein 7 | 0.21 | 72 | Collagen alpha-1(XII) chain | 0.26 |
| 73 | Lysozyme C | 0.21 | 73 | Ig heavy chain V-III region BUT | 0.26 |
| 74 | Apolipoprotein E | 0.20 | 74 | Ig kappa chain V-III region VG | 0.25 |
| 75 | Pyruvate kinase PKM | 0.18 | 75 | Ig kappa chain V-II region Cum | 0.25 |
| 76 | Hemopexin | 0.18 | 76 | Serum amyloid P-component | 0.25 |
| 77 | Extracellular superoxide dismutase [Cu-Zn] | 0.18 | 77 | Ig kappa chain V-III region VH | 0.25 |
| 78 | Alpha-2-HS-glycoprotein | 0.17 | 78 | Angiopoietin-related protein 7 | 0.24 |
| 79 | Haptoglobin-related protein | 0.17 | 79 | Tetranectin | 0.24 |
| 80 | Actin, cytoplasmic 1 | 0.17 | 80 | Ig kappa chain V-IV region | 0.23 |
| 81 | Fibromodulin | 0.16 | 81 | MAM domain-containing protein 2 | 0.23 |
| 82 | C-type lectin domain family 11 member A | 0.16 | 82 | 14-3-3 protein theta | 0.23 |
| 83 | Ferritin heavy chain | 0.16 | 83 | 14-3-3 protein zeta/delta | 0.23 |
| 84 | Prelamin-A/C | 0.16 | 84 | Annexin A1 | 0.22 |
| 85 | Keratin, type I cytoskeletal 12 | 0.15 | 85 | Glyceraldehyde-3-phosphate dehydrogenase | 0.22 |
| 86 | Coagulation factor XIII A chain | 0.15 | 86 | Histone H1.3 | 0.22 |
| 87 | Histone H1.3 | 0.15 | 87 | Peroxiredoxin-5, mitochondrial | 0.22 |
| 88 | Protein AMBP | 0.15 | 88 | Ig kappa chain V-II region RPMI 6410 | 0.21 |
| 89 | Keratin, type II cytoskeletal 1 | 0.14 | 89 | Galectin-7 | 0.21 |
| 90 | Keratin, type I cytoskeletal 14 | 0.13 | 90 | Tubulin alpha-1B chain | 0.18 |
| 91 | Serine protease HTRA1 | 0.13 | 91 | Phospholipase A2, membrane associated | 0.18 |
| 92 | Tubulin alpha-1B chain | 0.13 | 92 | Antithrombin-III | 0.18 |
| 93 | Antithrombin-III | 0.13 | 93 | Nucleoside diphosphate kinase A | 0.18 |
| 94 | Collagen alpha-1(II) chain | 0.13 | 94 | Annexin A5 | 0.17 |
| 95 | Complement C1q subcomponent subunit A | 0.13 | 95 | Thrombospondin-4 | 0.17 |
| 96 | Complement C1q subcomponent subunit B | 0.13 | 96 | Gelsolin | 0.17 |
| 97 | Beta-2-glycoprotein 1 | 0.11 | 97 | Fibromodulin | 0.16 |
| 98 | 14-3-3 protein epsilon | 0.11 | 98 | 40S ribosomal protein S10 | 0.16 |
| 99 | Collagen alpha-1(XI) chain | 0.11 | 99 | Pyruvate kinase PKM | 0.16 |
| 100 | Triosephosphate isomerase | 0.11 | 100 | Beta-2-glycoprotein 1 | 0.16 |
| 101 | Complement C4-A | 0.11 | 101 | Alpha-2-HS-glycoprotein | 0.15 |
| 102 | Fibrinogen alpha chain | 0.10 | 102 | Protein AMBP | 0.15 |
| 103 | Olfactomedin-like protein 3 | 0.10 | 103 | Apolipoprotein E | 0.14 |
| 104 | Cartilage acidic protein 1 | 0.10 | 104 | Histone H1.4 | 0.14 |
| 105 | Collagen alpha-1(VIII) chain | 0.10 | 105 | Microfibrillar-associated protein 2 | 0.14 |
| 106 | Complement component C8 beta chain | 0.10 | 106 | Haptoglobin | 0.13 |
| 107 | Complement factor H | 0.10 | 107 | Collagen alpha-1(II) chain | 0.13 |
| 108 | Ig mu chain C region | 0.09 | 108 | 60S ribosomal protein L15 | 0.12 |
| 109 | Alcohol dehydrogenase 1A | 0.08 | 109 | Prelamin-A/C | 0.12 |
| 110 | Olfactomedin-like protein 1 | 0.07 | 110 | Insulin-like growth factor-binding protein 6 | 0.12 |
| 111 | Transketolase | 0.07 | 111 | Keratin, type I cuticular Ha8 | 0.12 |
| 112 | Elongation factor 1-alpha 1 | 0.06 | 112 | Peroxiredoxin-6 | 0.12 |
| 113 | Fibulin-5 | 0.06 | 113 | Extracellular superoxide dismutase [Cu-Zn] | 0.11 |
| 114 | Keratin, type I cytoskeletal 15 | 0.06 | 114 | Keratin, type II cytoskeletal 1 | 0.11 |
| 115 | Tubulin alpha-1A chain | 0.06 | 115 | Alpha-1B-glycoprotein | 0.11 |
| 116 | Tubulin beta-4A chain | 0.06 | 116 | Collagen alpha-2(VIII) chain | 0.11 |
| 117 | Histidine-rich glycoprotein | 0.06 | 117 | Ig mu chain C region | 0.10 |
| 118 | Kininogen-1 | 0.05 | 118 | Keratin, type I cytoskeletal 9 | 0.10 |
| 119 | Thrombospondin-1 | 0.05 | 119 | NAD(P)H dehydrogenase [quinone] 1 | 0.10 |
| 120 | Complement factor B | 0.04 | 120 | Collagen alpha-1(VIII) chain | 0.10 |
| 121 | Complement C5 | 0.02 | 121 | Coagulation factor XIII A chain | 0.09 |
|  |  |  | 122 | Hemopexin | 0.09 |
|  |  |  | 123 | Collagen alpha-1(XI) chain | 0.08 |
|  |  |  | 124 | Complement C4-A | 0.08 |
|  |  |  | 125 | Kininogen-1 | 0.08 |
|  |  |  | 126 | Thrombospondin-1 | 0.08 |
|  |  |  | 127 | Fibulin-5 | 0.08 |
|  |  |  | 128 | Complement factor H | 0.07 |
|  |  |  | 129 | Complement component C8 beta chain | 0.06 |
|  |  |  | 130 | Tubulin beta-4A chain | 0.05 |
|  |  |  | 131 | Fibrinogen gamma chain | 0.05 |
|  |  |  | 132 | Extracellular matrix protein 1 | 0.05 |
|  |  |  | 133 | Fibulin-1 | 0.05 |
|  |  |  | 134 | Complement component C8 alpha chain | 0.04 |
|  |  |  | 135 | Alpha-2-macroglobulin | 0.03 |
|  |  |  | 136 | Collagen alpha-3(IV) chain | 0.03 |
|  |  |  | 137 | Inter-alpha-trypsin inhibitor heavy chain H4 | 0.03 |
|  |  |  | 138 | Metabotropic glutamate receptor 4 | 0.03 |
|  |  |  | 139 | Periostin | 0.03 |
|  |  |  | 140 | Laminin subunit beta-3 | 0.02 |
|  |  |  | 141 | Desmoplakin | 0.01 |
|  |  |  | 142 | Neuroblast differentiation-associated protein AHNAK | 0.01c |
